# Coupling Novel Probes with Molecular Localization Microscopy Reveals Cell Wall Homeostatic Mechanisms in *Staphylococcus aureus*

**DOI:** 10.1021/acschembio.2c00741

**Published:** 2022-11-22

**Authors:** Victoria A. Lund, Haneesh Gangotra, Zhen Zhao, Joshua A. F. Sutton, Katarzyna Wacnik, Kristen DeMeester, Hai Liang, Cintia Santiago, Catherine Leimkuhler Grimes, Simon Jones, Simon J. Foster

**Affiliations:** † School of Biosciences, University of Sheffield, Sheffield S10 2TN, United Kingdom; ‡ The Florey Institute for Host−Pathogen Interactions, University of Sheffield, Sheffield S10 2TN, United Kingdom; § The Department of Chemistry, University of Sheffield, Sheffield S3 7HF, United Kingdom; ∥ Department of Chemistry and Biochemistry and Department of Biological Sciences, University of Delaware, Newark, Delaware 19716, United States

## Abstract

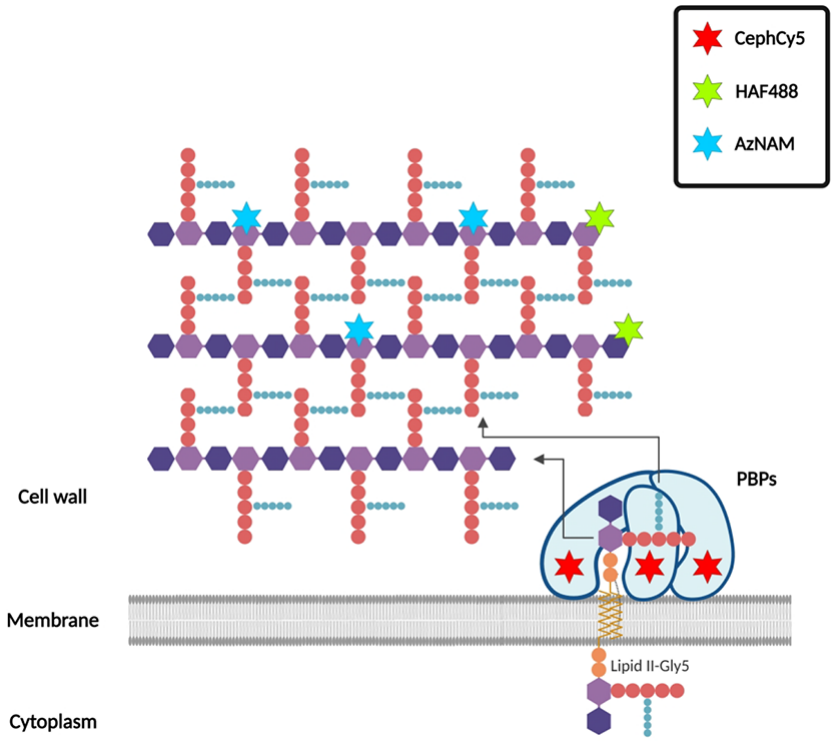

Bacterial cell wall peptidoglycan is essential for viability, and its synthesis is targeted by antibiotics, including penicillin. To determine how peptidoglycan homeostasis controls cell architecture, growth, and division, we have developed novel labeling approaches. These are compatible with super-resolution fluorescence microscopy to examine peptidoglycan synthesis, hydrolysis, and the localization of the enzymes required for its biosynthesis (penicillin binding proteins (PBPs)). Synthesis of a cephalosporin-based fluorescent probe revealed a pattern of PBPs at the septum during division, supporting a model of dispersed peptidoglycan synthesis. Metabolic and hydroxylamine-based probes respectively enabled the synthesis of glycan strands and associated reducing termini of the peptidoglycan to be mapped. Foci and arcs of reducing termini appear as a result of both synthesis of glycan strands and glucosaminidase activity of the major peptidoglycan hydrolase, SagB. Our studies provide molecular level details of how essential peptidoglycan dynamics are controlled during growth and division.

The bacterial cell wall is essential for life and determines cell morphology.^1^ The major cell wall structural polymer is peptidoglycan (PG), which as a single macromolecule surrounding the cell resists internal turgor.^2^ PG biosynthesis is also the target of crucial antibiotics such as the β-lactams and vancomycin.^2,3^ PG has a dynamic structure allowing cell growth and division, while maintaining its integrity. Peptidoglycan consists of glycan chains of repeating *N-*acetylglucosamine (Glc*N*Ac) and *N*-acetylmuramic acid (Mur*N*Ac) disaccharides cross-linked with peptides that are diverse in composition across different bacterial species.^2^ Synthesis of peptidoglycan requires the concerted action of multiple enzymes, including the cytoplasmic synthesis of precursors, transfer across the cytoplasmic membrane, and final incorporation into the existing cell wall. Postsynthesis, peptidoglycan remains a dynamic structure through modifications, the addition of other components, and hydrolysis.^4^ The culmination of all of these processes leads to a complex peptidoglycan architecture that has only just begun to be elucidated at the single-molecule level, primarily using atomic force microscopy.^5,6^ Difficulties in determining peptidoglycan architecture have been in large part due to the necessity to image the material *in situ* as the single cell sacculus or in live cells. We have recently revealed that the cell wall peptidoglycan of the important human pathogen *Staphylococcus aureus* is an expanded hydrogel in which the mature material presents with an external surface pitted by holes but an internal surface of a much smoother appearance with small pores.^6^ Also, during division, a further architecture is present with the newly exposed septal surface having rings, with individual glycan strands being visible. This ring structure matures during the cell cycle into the pitted mesh. These architectural dynamics allude to several mechanisms of coordinated synthesis and hydrolysis leading to the observed features.

The final stage in the synthesis of peptidoglycan, outside the cytoplasmic membrane, includes the penicillin binding proteins (PBPs) that add new muropeptides into the existing cell wall through transglycosylase and transpeptidase activities.^7^ PBPs can be subdivided into class A PBPs, which are bifunctional, class B PBPs, which possess transpeptidase activity and low molecular weight, which mostly have d,d-carboxypeptidase activity.^8,9^*S. aureus* has four endogenous PBPs, of which PBP1 and PBP2 are the only essential enzymes.^10^ PBP1 has a specific role in cell division, while PBP2 is more generalized. Recently, FtsW has been found to have transglycosylase activity and form a cognate pair with PBP1 in cell division.^11^ FtsW is a so-called SEDS protein, the other being RodA (also shown to have transglycosylase activity) that partners with PBP3 with a role in peripheral cell wall synthesis.^11^ PBP4 has a function in cross-linking of the peptidoglycan during cell division.^12^ Finally, there are two monofunctional transglycosylases (Mgt, SgtA) with nonessential roles in cell wall synthesis.^13^ As well as multiple PG synthesis components, *S. aureus* has many PG hydrolases, mostly of unknown and likely overlapping function.^14,15^ Having so many players, coupled with them being involved in determining the growth and division of a spheroid bacterium only 1 μm in diameter makes analysis of their role in cell wall dynamics difficult. The advent of high resolution AFM has begun to set the architectural scene for their functions.^6^ However, for further understanding, we must correlate high resolution PG architecture with determination of the localization and dynamics of the components involved and mapping their activities at the single cell level. This requires a combination of super-resolution microscopy and the development of specific probes. At the forefront of this approach with PG synthesis has been the development of fluorescent d-amino acid (FDAA) derivatives that have given a step change in our understanding of PG synthesis *in vivo*.^16−18^ The FDAAs give a proxy for PG synthesis as they are incorporated into the existing sacculus. More recently, metabolic probes for glycan synthesis have been developed, providing complementary information to FDAAs.^19,20^ The single-molecule microscopy approach of stochastic optical reconstruction microscopy (STORM) has been applied to *S. aureus*, demonstrating that PG is synthesized at the septum and around the peripheral wall.^18^ During cell division, PG synthesis, as measured by FDAA incorporation, was found to be dispersed across the developing septal surface, a pattern that matches the morphology of the septum as “V” shaped before filling the annulus and then bowed afterward, finally becoming parallel sided before cell separation.^18^ After cell separation the newly exposed external peptidoglycan ring structure matures during the cell cycle, due to the action of peptidoglycan hydrolases allowing cell expansion. Thus, observable cell cycle associated morphological events can begin to be explained by molecular level mechanisms of peptidoglycan synthesis and hydrolysis.

Here, we have used three complementary probes to be able to study peptidoglycan dynamics, at the molecular level in *S. aureus*, based on an existing technique to analyze glycan synthesis and two novel approaches to map the peptidoglycan biosynthetic machinery and glycan strand length dynamics. Coupling these approaches with single molecule, super-resolution fluorescence microscopy has revealed a coordinated molecular framework of peptidoglycan synthesis and hydrolysis that together control cell morphological flux through the cell cycle.

**Distribution of Penicillin Binding Proteins.** The final stage of peptidoglycan biosynthesis requires the action of PBPs, and so they form the nexus between growth and division components and the cell wall peptidoglycan product. Single molecule PBP localization is difficult as PBPs are often present in low abundance and there is a requirement for fluorophores compatible with technologies such as STORM. Cephalosporin antibiotics can show specificity to individual PBPs and have also been shown to be amenable to fluorescently labeled PBPs.^21^ Here, we developed a set of new fluorescent cephalosporins by ligating novel scaffolds to various fluorophores, prior to evaluating their potential for the biological imaging of PBPs. Subsequently, CephCy5 was chosen for this study due to its compatibility with STORM (Figure 1a and Figure S1). CephCy5 labels PBP1, 2, and 3 *in vitro*, unlike Bocillin-Fl, which labels all four PBPs in *S. aureus*, but both Bocillin and CephCy5 have PBP2 most prominently labeled (Figure 1b). Importantly, in addition, CephCy5 maintains antibiotic properties *in vivo* with an MIC of 20 μg/mL, compared to 5 μg/mL for the parental cephalosporin. CephCy5 labeling of whole cells in *S. aureus* SH1000 gives a localization consistent with that of PBPs in the cell membrane (Figures 1c, S1a). The antibiotic action of CephCy5 has no effect on localization since labeling of cells both pre- and post-fixation shows the same localization pattern (Figure S1b). An excess of methicillin in the labeling reaction leads to a significant decrease in CephCy5 labeling (Figure S1c) underlining the specificity of CephCy5 for the PBP active site.

**Figure 1:**
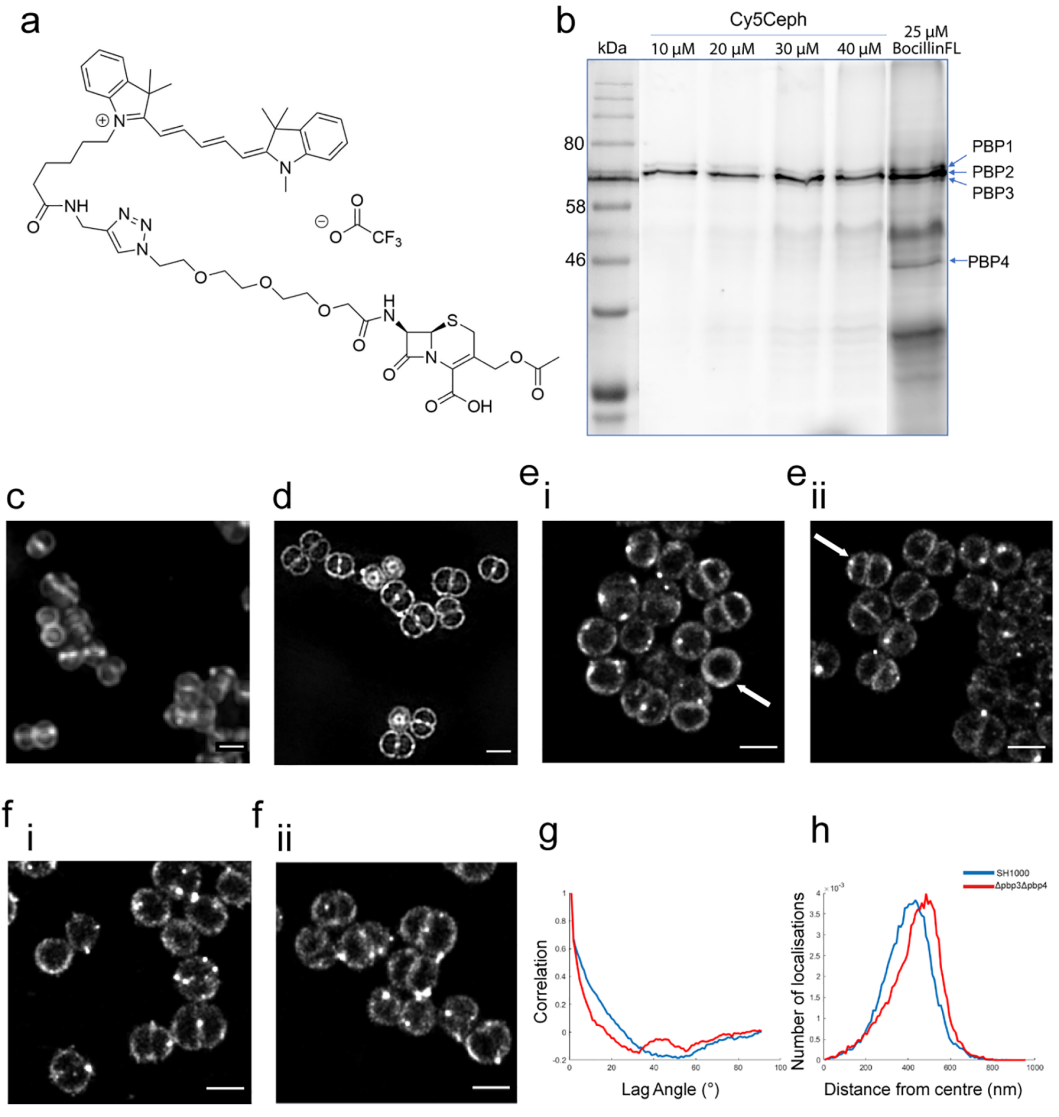
Molecular localization of PBPs using a novel β-lactam probe. (a) Structure of CephCy5. (b) SDS-PAGE of membrane proteins from SH1000 labeled with increasing concentrations of CephCy5 or BocillinFl. (c–e) Microscopy images of *S. aureus* SH1000 labeled with CephCy5. (c) Deconvolution widefield microscopy, scale bar = 1 μm. (d) SIM microscopy, scale bar = 1 μm. (e.i and e.ii) STORM microscopy, scale bar = 1 μm. There were no clear foci; however, there does appear to be some regions with an increased number of localizations, suggesting an accumulation of PBPs in these areas (example septa Figure 1e.i marked with an arrow). (f.i,ii) Representative dSTORM images of *S. aureus* SH4424 (*pbp3 pbp4*) labeled with CephCy5. Scale bar = 1 μm. (g) Autocorrelation of angles of distribution of localizations for CephCy5 labeled *S. aureus* SH1000 (blue line) and SH4424 (*pbp3 pbp4*, red line), *n* = 10 septa. (h) Average distance of localizations from the center of developing septa for the same samples, *n* = 10 septa.

CephCy5 labeling, coupled with diffraction limited microscopy or structured illumination microscopy (SIM), revealed the PBPs to be present both at the septum and at the cell periphery, consistent with PG biosynthesis occurring in both regions. 3D-SIM also suggests that during septum formation, PBPs may be found at a higher concentration at the leading edge of the septum (Figure 1d). The septal leading edge is at its apex where PG is actively being synthesized to fill the annulus.^18^ However, it is known that 3D-SIM can introduce imaging artifacts.^22^ We therefore used direct stochastic optical reconstruction microscopy (dSTORM) to give molecular level analysis of PBP localization. Localizations can be observed throughout the cell membrane, both at the septum and at the periphery (Figure 1e). There were no clear foci; however, there do appear to be some regions with an increased number of localizations, suggesting an accumulation of PBPs in these areas (example septa Figure 1e.i marked with an arrow). In cells with a septal plate perpendicular to the plane of imaging, a clear gap is visible, this has a measurement of approximately 150 nm (*n* = 6, SD 26.08; example septum Figure 1e.ii marked with an arrow). This corresponds to the presence of septal peptidoglycan, and indeed two color images of *N*-hydroxysuccinimide (NHS) Ester-Atto 488, which labels amine groups present in the cell wall,^18,23^ and CephCy5-labeled SH1000 show that PBPs are localized inside the cell wall material (Figure S1a, a cell demonstrating this has been highlighted with an arrow), as would be expected. Single molecule PBP localization across multiple septa, in the plane of focus, was analyzed using our previously devised methods.^18^ A circle is fitted to the experimental data, and each individual localization is assigned an angle and distance from the center of the fitted circle (Figure S2). The angular distribution of the PBPs shows that these have neither completely random nor regular distribution around the septal ring (Figure 1g), as we have previously observed for cell division components.^4^ Therefore, while there is some level of order of PG synthesis enzymes, there is no evidence of an exact number of macromolecular complexes within the septum. The STORM data do not show an accumulation of PBPs at the leading edge of the septa (Figure 1h); indeed PBPs are distributed across all the septal material. This is in agreement with previously published FDAA data, which showed that the product of PBP reactions was laid down across the cell septum.^18^

To determine if any of the observed labeling was due to the nonessential PBPs, a strain that lacked both PBP3 and PBP4 (SH4424 - *S. aureus* SH1000 *pbp3::spec pbp4::Tn*) was created. dSTORM of CephCy5 labeled SH4424 cells revealed PBP localization comparable to the SH1000 parent (Figures 1f,g,h; S1e,f; S2).

**Glycan Chain Remodelling by Glucosaminidase Activity.** The PG of *S. aureus* has a complex and dynamic architecture as a result of synthesis and hydrolysis. The length of the glycan strands is a key determining feature and varies widely between bacteria.^5,24,25^ The glycan strands in *S. aureus* are synthesized as long chains and then cleaved by glucosaminidase enzymes.^24^ The glucosaminidases are required for cell growth, and loss of the major enzyme (SagB) results in longer glycans, increased wall stiffness, and an alteration in observed PG architecture.^15,6^ Reducing termini form the ends of newly synthesized glycan strands and appear as a result of glucosaminidase activity.^2^ Thus, in the glycan chain, reducing termini dynamics provide a window into the appearance of new glycans and their modification during growth and division. The carbonyl group of the reducing termini is unique in peptidoglycan chemistry and provides a target for fluorescent labeling via aldehyde ligation to hydroxylamine. Thus, when commercial fluorophore-conjugated hydroxylamine (AlexaFluor488-Hydroxylamine = HAF488) is used, reducing termini can be imaged.

Treating SH1000 cells with HAF488 produces a cell wall labeling pattern with a clear focus at the site of cell division (Figure 2a, d, e, and f). HAF488 labeling is specific to reducing termini as it is abolished by the addition of the reducing agents, sodium borohydride (NaBH_4_), or unfunctionalized hydroxylamine (Figure S3a–c). Also, HAF488 binds to peptidoglycan, as extracted and purified *S. aureus* sacculi are found to be labeled (Figure S3d).

**Figure 2:**
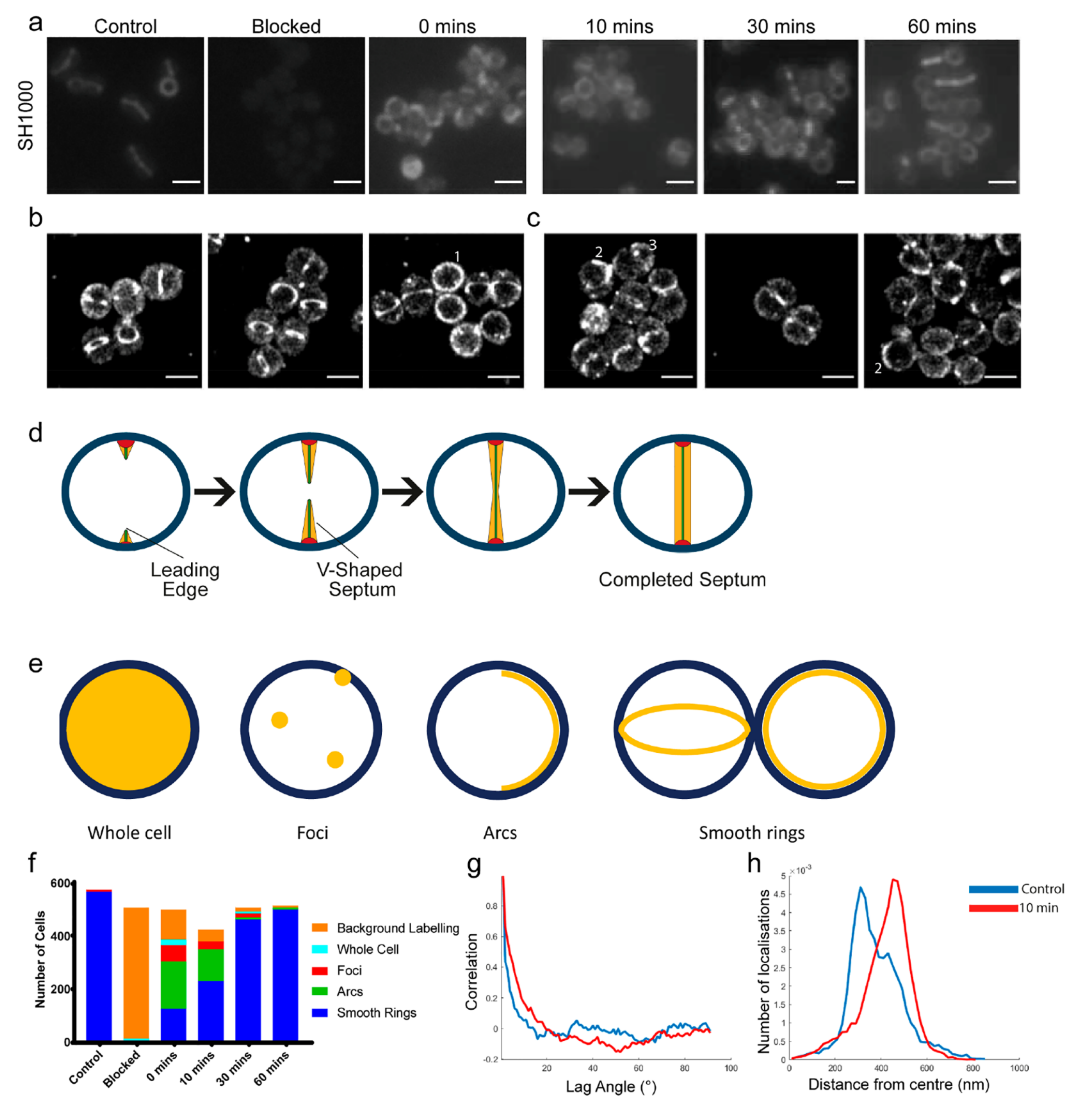
Molecular localization of cell wall reducing termini. (a) Time course of the appearance of reducing termini (labeled with HAF488) in *S. aureus* SH1000 following blocking with hydroxylamine (*T* = 0–60 min, time after resuspension in growth media). Scale bar 1 μm. (b) Three representative dSTORM images of SH1000 labeled with AzTEG 647. Scale bar = 1 μm. (c) Three representative dSTORM images of SH1000 labeled with AzTEG 647 after 10 min regrowth following blocking with hydroxylamine. Scale bar = 1 μm. (d) *S. aureus* septum formation begins with the production of the PG “piecrust” (red). The septum then develops as a V-shaped septal plate until the annulus fuses, resulting in a bowed morphology. PG is then inserted until a uniform septal thickness is achieved. (e) Labeling patterns produced by HAF488 in *S. aureus.* HAF488 (yellow) can be found uniformly across the whole cell (blue) or in discrete foci. HAF488 could also be found in arcs, as well as in smooth rings (two orientations shown). (f) Analysis of HAF488 labeling pattern over time following blocking with hydroxylamine. *n* = ∼550 per condition. Numbers in b and c show representative labeling patterns. 1, smooth rings; 2, arcs; and 3, foci. (g) Autocorrelation of angles of distribution of localizations for AzTEG 647 labeled samples in b and c, *n* = 5 septa. (h) Average distance of localizations from the center of developing septa for AzTEG 647 labeled samples from b and c, *n* = 5 septa (control blue line, 10 min red line).

Reducing termini in *S. aureus* are located primarily at the septum, while there is some labeling throughout the rest of the cell (Figure 2a). Septal labeling accounts for about 50% of the total cell fluorescence (Figure S3e). To determine the dynamics of the appearance of reducing termini, existing reducing termini were blocked using hydroxylamine, before *S. aureus* cells were washed and resuspended in growth media. Reducing termini reappear almost immediately on the removal of the blocking agent and resuspension in growth media (Figures 2a and S3f). However, the pattern of reducing termini labeling evolves over time at the septum with the initial appearance of foci and arcs (Figure 2f). Quantification of labeling patterns demonstrates that immediately following blocking, 45% of cells have arcs or foci, while 25% have septal rings compared to almost 100% rings in the control (Figure 2f). Within 30 min, the labeling pattern has developed to >90 rings (Figure 2f). After blocking, the generation of reducing termini is determined by either the synthesis of new glycan chains containing a reducing terminus or the hydrolysis of existing chains. SagB is known to be the major PG hydrolase responsible for glycan chain length determination.^13^ Glycan strand synthesis is essential for growth, but the two monofunctional transglycosylases (Mgt and Sgt) are not.^13^ To determine the roles of these components in reducing termini, generation strains that lacked the monofunctional transglycosylases and SagB were studied (SH4644 *mgt sgtA*, SH4608 *sagB*, and SH4659 *mgt sgtA sagB*; Figure S4). Visually, there is no apparent difference between these strains, and there is no significant alteration in the level of labeling between strains (Figure S4). However, both strains that lacked the glucosaminidase, SagB, took longer for the control labeling morphology to appear after blocking of reducing termini (Figure S4). At 30 min after blocking, strain SH4608 (*sagB*) showed ∼65% control morphology compared with ∼90% for SH1000.

To demonstrate the molecular level localization of reducing termini, a novel dSTORM compatible probe, 1-[2-(aminooxy)ethoxy]-2-(2-azidoethoxy)ethane,^26^ preclicked with Alexafluor 647 (AzTEG 647) was prepared. Cells were labeled with AzTEG 647 without prior treatment (Figure 2b) or after 10 min of recovery, post hydroxylamine blocking (Figure 2c), before imaging using dSTORM. The untreated sample shows a high density of localizations at the septum compared to the postblocking, 10 min recovery sample. Analysis of the localization of events in the septum showed no obvious patterning for either sample (Figures 2g and S5a,b). However, there is a bias of localizations toward the leading edge of the septum in the control compared to the 10 min recovered sample (Figures 2h and S5b).

**Synthesis of Nascent Glycan Chains.** Peptidoglycan synthesis has previously been localized through the use of FDAAs that label the peptide side chains in nascent material.^16,17,27,18^ However, the other defining feature of biosynthesis is that of the glycan strands. Recent work has shown that it is possible for bacteria to insert modified MurNAc sugars through the peptidoglycan recycling pathway native to *Pseudomonas putida*.^19,20^ This provided a framework to build an equivalent experimental system in *S. aureus* to analyze glycan synthesis. *S. aureus* has several transglycosylases, including the monofunctional enzymes Mgt and SgtA, and the recently described SEDs proteins (FtsW and RodA).^11,13^ To dissect the individual and combined roles of these enzymes on the cellular and molecular levels requires the ability to be able to measure glycan synthesis *in situ*, in the living cell. To expedite this process, we expressed the MurNAc/GlcNAc anomeric kinase (*amgK*) and NAM α-1 phosphate uridylyl transferase (*murU*) from *P. putida* under the control of a constitutive promoter in *S. aureus* (SH5097). This allowed incorporation of azide *N*-acetylmuramic acid (AzNAM),^19,20^ into the developing peptidoglycan structure (Figure 3). Labeling SH5097 (S. *aureus* SH1000 *geh:: P*_*pcn*_*-amgK-murU*) with AzNAM for 5 min allowed visualization of glycan synthesis by deconvolution, SIM, and dSTORM microscopy. Across the imaging modalities, AzNAM labeling could be seen throughout the cell wall, indicating synthesis at the septum and cell periphery (Figure 3). In order to determine the pattern of AzNAM incorporation and therefore glycan synthesis, the labeling time was reduced to less than 5 min as we have done previously for FDAA. However, this resulted in insufficient labeling to be able to make an analysis (Figure S6), suggesting that the biorthogonal labeled probe cannot compete with unlabeled NAM on this time scale. The development of metabolic glycan labeling for *S. aureus* is a useful addition to our repertoire for, for instance, effects of inhibitors but will require further refinement in order to give sufficient incorporation to compare to the FDAA molecular localization after seconds of addition.^18^

**Figure 3:**
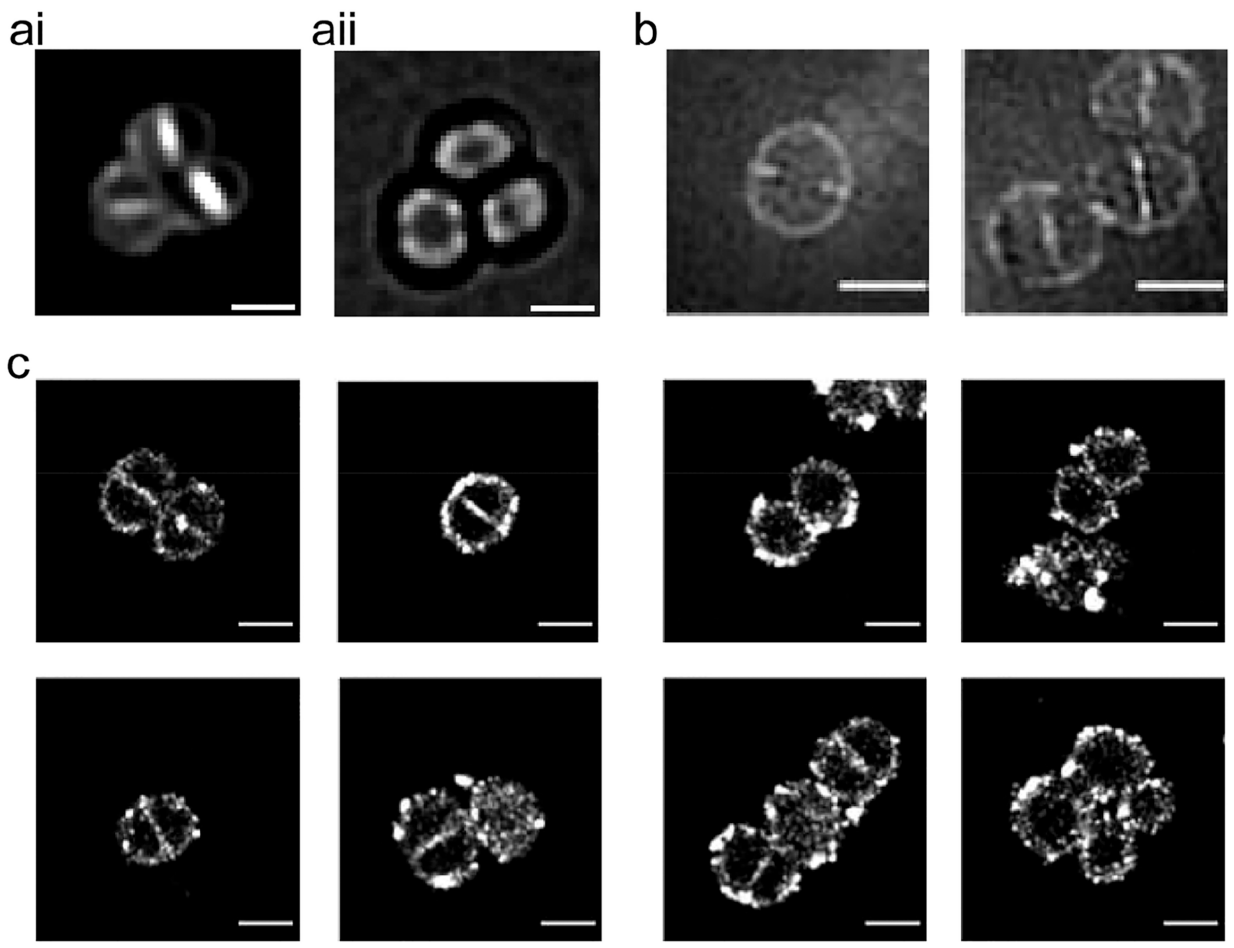
Molecular localization of glycan strand synthesis. SH5097 (S. *aureus* SH1000 *geh:: P*_*pcn*_*-amgK-murU*) was labeled for 5 min with AzNAM clicked to Atto-488 (deconvolution and SIM) or AlexaFluor647 (dSTORM). (a.i) Deconvolution of AzNAM Atto-488. (a.ii) Corresponding DIC image of a.i, scale bar = 1 μm. (b) SIM, scale bar = 1 μm. (c) dSTORM, scale bar = 500 nm.

Bacterial cell wall dynamics underpins growth and division, requiring the coordinated activity of multiple synthesis and hydrolysis components. The functions of these components are manifested within the spatial context of the cell, and so it is necessary to be able to view the localization and activities of the wall dynamics components *in situ*. This has proven to be very difficult due to the diminutive size of bacteria and the resolution of microscopy approaches. Only recently are new imaging modalities and functional probes beginning to elucidate these complex mechanisms. The FDAA system developed by Kuru et al. as proxy for the transpeptidase activity of the PBPs has proved seminal and has been utilized by many groups to reveal where PG synthesis has occurred.^16,28^

Determining the spatial distribution of PBPs demonstrates where these crucial enzymes are located.^29,30^ Here, we have developed a new chemical probe, based on cephalosporin, that maintains activity as shown by its ability to inhibit growth and gives labeling compatible with the molecular level resolution of dSTORM. The specificity of the probe revealed that it is able to label PBP2 as its main target. Potential labeling by nonessential PBPs (PBP3 and 4) was ruled out using appropriate genetically mutated strains, which is important as previous research has shown that PBP4 can synthesize PG at the peripheral wall.^31^ PBP2 is an essential PBP with both transpeptidase and transglycosylase activity.^10^ Previous, lower resolution studies revealed PBP2 to be primarily located at the cell septum.^32^ Here, the CephCy5 label gave a localization pattern at both the septum and cell periphery alluding to the role of PBP2 at both of these sites. We have previously found that PG synthesis at the cell periphery occurs even in the absence of PBP4, showing that PBP2 is the primary enzyme with this function, as PBP1 is apparently septum formation specific.^11,18^ There has been extensive analysis of the mode of septal synthesis in *S. aureus* and many other bacteria, previously suggesting that the division apparatus, the divisome, forms a ring at the leading edge of the developing septum.^32,33^ Recently, our super-resolution studies using FDAA labeling and fluorescent reporters with cell division components have resulted in a novel model for division that is also based on the observed septal morphology.^18^ The developing septum in *S. aureus* is V shaped (Figure S3a), giving a large surface area for concomitant synthesis. Here, labeling the enzymes directly responsible of PG synthesis gave a similar diffuse pattern, supporting a more dispersed model for septum formation.

As cell wall dynamics are controlled by synthesis and hydrolysis, we developed a labeling modality that could capture both of these features. As well as the level of cross-linking, another PG defining feature is the length of the glycan strands. We exploited the reducing termini at the end of glycan strands as a probe ligand. We used a combination of a commercially available fluorescent label (HAF488) and generated an azido-hydroxylamine probe (AzTEG 647), as a novel probe for super resolution imaging. The specificity of the probe was verified by isolated sacculi labeling and the ability to block the reducing termini. This blocking phenomenon also allowed the dynamic appearance of reducing termini to be investigated during growth. Reducing termini are present throughout the cell wall, at both the septum and periphery. After blocking, an uneven evolution of reducing termini occurs with distinct foci and arcs appearing at the septum before complete labeling appears. *S. aureus* is characterized by having short glycans in the mature PG as a result of the activity of the glucosaminidase, SagB.^15,24^ Loss of SagB led to a delayed rate of reducing termini formation in foci and arcs, supporting our previous assertion that this enzyme is involved in PG maturation. This also suggests that SagB is active at the septum, which we have previously hypothesized as a loss of this enzyme resulting in a delayed maturation of the newly exposed septum after cell separation.^6^ Molecular localization of reducing termini revealed the appearance not only of foci and arcs but also in many cases a ring of labeling at the leading edge of the developing septum. Reducing termini are the result of both synthesis and hydrolysis giving a pattern not observed for just synthesis alone (FDAA) or synthesizing enzymes (CephCy5).

To complete the picture for major PG chemical features via labeling approaches, we adapted one recently published for metabolic labeling of glycan strands.^19^ This required expressing those enzymes necessary for a peptidoglycan recycling pathway native to *Pseudomonas putida* in *S. aureus*.^19,20^ The constructed strain provides a stable framework for analysis of glycan strand biosynthesis in *S. aureus.* However, the level of incorporation achieved was not sufficient to permit the analysis of the molecular localization of glycan synthesis in *S. aureus* at the low time scales necessary for molecular level incorporation studies. Future work is needed to determine how to bolster incorporation rates in *S. aureus* without the use of antibiotics.

By using a combination of microscopy approaches, the same phenomenon can be observed in differing levels of detail. Widefield microscopy uses lower light intensities than other methods, allowing fluorophores that easily bleach to be viewed and at a higher throughput, albeit at lower resolution, than other techniques. SIM provides a higher resolution and is useful for imaging a combination of fluorophores simultaneously but can lead to signal bleaching due to the high level of light exposure required. The highest resolution is provided by STORM, which gives single molecule representation, but the data can be difficult to contextualize in the absence of multiple labels.

In order to unravel the intricacies of bacterial cell growth and division, we must employ a range of complementary approaches to determine molecular events within the context of the live cell. It is by an active development of both underpinning imaging modalities, matched with a suite of specific probes, that we are beginning to reveal the life of bacteria.

## Supporting Information Available

Tables containing strains, plasmids, and oligonucleotides; further microscopy utilizing CephCy5 with dSTORM determined localizations; analysis of HAF488 localization; localization of AzTEG 647 associated with septa by dSTORM; microsocopy of cells labeled with AzNAM; generic materials, including vendor information, and methodology; synthesis methods and confirmation of probes used (PDF)
