# Supplementary material for: Coupling Novel Probes with Molecular Localization Microscopy Reveals Cell Wall Homeostatic Mechanisms in Staphylococcus aureus
Source: ACS Chem Biol. 2022 Nov 22;17(12):3298–305. doi: 10.1021/acschembio.2c00741 (PMC9764285; doi:10.1021/acschembio.2c00741)
Supplement: Supplementary file 1 — cb2c00741_si_001.pdf [file cb2c00741_si_001.pdf]

Supporting Information for

Coupling novel probes with molecular localisation microscopy reveals cell wall homeostatic mechanisms in Staphylococcus aureus

Victoria A Lund[1,2#], Haneesh Gangotra[2,3#], Zhen Zhao[2,3#], Joshua A F Sutton[1,2#], Katarzyna Wacnik[1,2], Kristen DeMeester[4], Hai Liang[4], Cintia Santiago[4], Catherine Leimkuhler Grimes[4], Simon Jones[2,3*], Simon J Foster[1,2*]

[1] School of Biosciences, University of Sheffield, Sheffield, S10 2TN, UK. [2] The Florey Institute for Host-Pathogen Interactions, University of Sheffield, Sheffield, S10 2TN, UK. [3] The Department of Chemistry, University of Sheffield, Sheffield, S3 7HF, UK. [4] Department of Chemistry and Biochemistry and Department of Biological Sciences, University of Delaware, Newark, Delaware 19716, United States

[#] These authors contributed equally

[*] To whom correspondence should be sent. Email : simon.jones@sheffield.ac.uk, s.foster@sheffield.ac.uk

Table of Contents

**Table S1.** Strains used in this study	S3
**Table S2.** Plasmids used in this study	S5
**Table S3.** Oligonucleotides used in this study	S5
**Figure S1:** CephCy5 labels PBPs in *S. aureus.*	S6
**Figure S2:** Analysis of CephCy5 molecular localisations.	S8
**Figure S3:** HAF488 labelling of reducing termini.	S9
**Figure S4:** Role of cell wall homeostasis components in molecular localisation of reducing termini.	S10
**Figure S5:** Analysis of HAF-linker molecular localisations.	S11
**Figure S6:** Labelling glycan strand synthesis using AzNAM.	S12
Materials and Methods	S13
Synthetic Methods and Characterization	S17
References	S23

Table S1. Strains used in this study

Strain	Relevant Genotype/markers	Source
SH1000	Functional *rsbU*$^{+}$ derivative of 8325-4	[1]
CYL316	*S. aureus* RN4220 pCL122Δ19; CmR	[2]
NE420	*S. aureus* JE2 with transposon insertion in *pbp3::Tn*; EryR	[3]
NE3003	*S. aureus* RN4220 pSPC; SpecR	[3]
SH4421	*S. aureus* SH1000 *pbp3::Tn*; EryR	This Study
SH4422	*S. aureus* SH1000 *pbp3::spec*; SpecR	This Study
SH4424	*S. aureus* SH1000 *pbp3::spec pbp4::Tn*; SpecR, EryR	This Study
SH4425	*S. aureus* SH1000 *pbp4::Tn*; EryR	[4]
SH4608	*S. aureus* SH1000 *sagB::kan*; KanR	[5]
NE596	*S. aureus* JE2 with transposon insertion in *mgt::Tn*; EryR	[3]
NE267	*S. aureus* JE2 with transposon insertion in *sgtA::Tn*; EryR	[3]
SH4628	*S. aureus* SH1000 *mgt::Tn*; EryR	This Study
SH4629	*S. aureus* SH1000 *sgtA::Tn*; EryR	This Study

NE3003	*S. aureus* RN4220 pTET; TetR	[3]
SH4643	*S. aureus* SH1000 *sgtA::tet*; TetR	This Study
SH4644	*S. aureus* SH1000 *mgt::Tn sgtA::tet*; EryR, TetR	This Study
SH4659	*S. aureus* SH1000 *mgt::Tn sgtA::tet sagB::kan*; EryR, TetR, KanR	This Study
SH4969	*S. aureus* RN4220 *geh::* P_{pcn}-*amgK-murU*; TetR	This Study
SH5097	*S. aureus* SH1000 *geh::* P_{pcn}-*amgK-murU*; TetR	This Study

Table S2. Plasmids used in this study

Plasmid	Relevant Genotype/markers	Source
pKASBAR-P_{pcn}*amgK murU*	pKASBAR-Tet containing *amgK* & *murU* under the *pcn* promoter; AmpR, TetR	This Study.

Table S3. Oligonucleotides used in this study.

**Oligonucleotide name**	**Sequence (5' to 3')***	**Use**	**Source**
AmgKMurU_pKASBAR_F	cagctatgaccatgattacgtgacccgcttgacggctag	Amplifies the *AmgKMurU* construct for insertion into pKASBAR by Gibson assembly.	This study
AmgKMurU_pKASBAR_R	ctgccctttttttgccccggtcaggcgcgctcgccaat		This study
PBP3-Tn-F	tgatgaaaacattacagtgaatg	Amplify the *pbp3* locus	This study
PBP3-Tn-R	gtatcgccatatggatattttc		This study
pbp4-1	ctgcagaaaactttattttc aac	Amplify the *pbp4* locus	[6]
pbp4-5	tatatagaactatcgatac taaac		[6]
Mgt_F	aaaccttctacattatagttcc	Amplify the *mgt* locus	This study
Mgt_R	ctcaaggtatatactaagtgag		This study
SgtA_F	gtcaaattgcttaaatatggtata	Amplify the *sgtA* locus	This study
SgtA_R	atagcatatgtatgattaactttg		This study
sagB_F	ccgatcagatatttttcaaa ca	Amplify the *sagB* locus	[5]
sagB_R	cttattcaaatgtttactgt catc		[5]
*Overhangs for the Gibson assembly in blue			

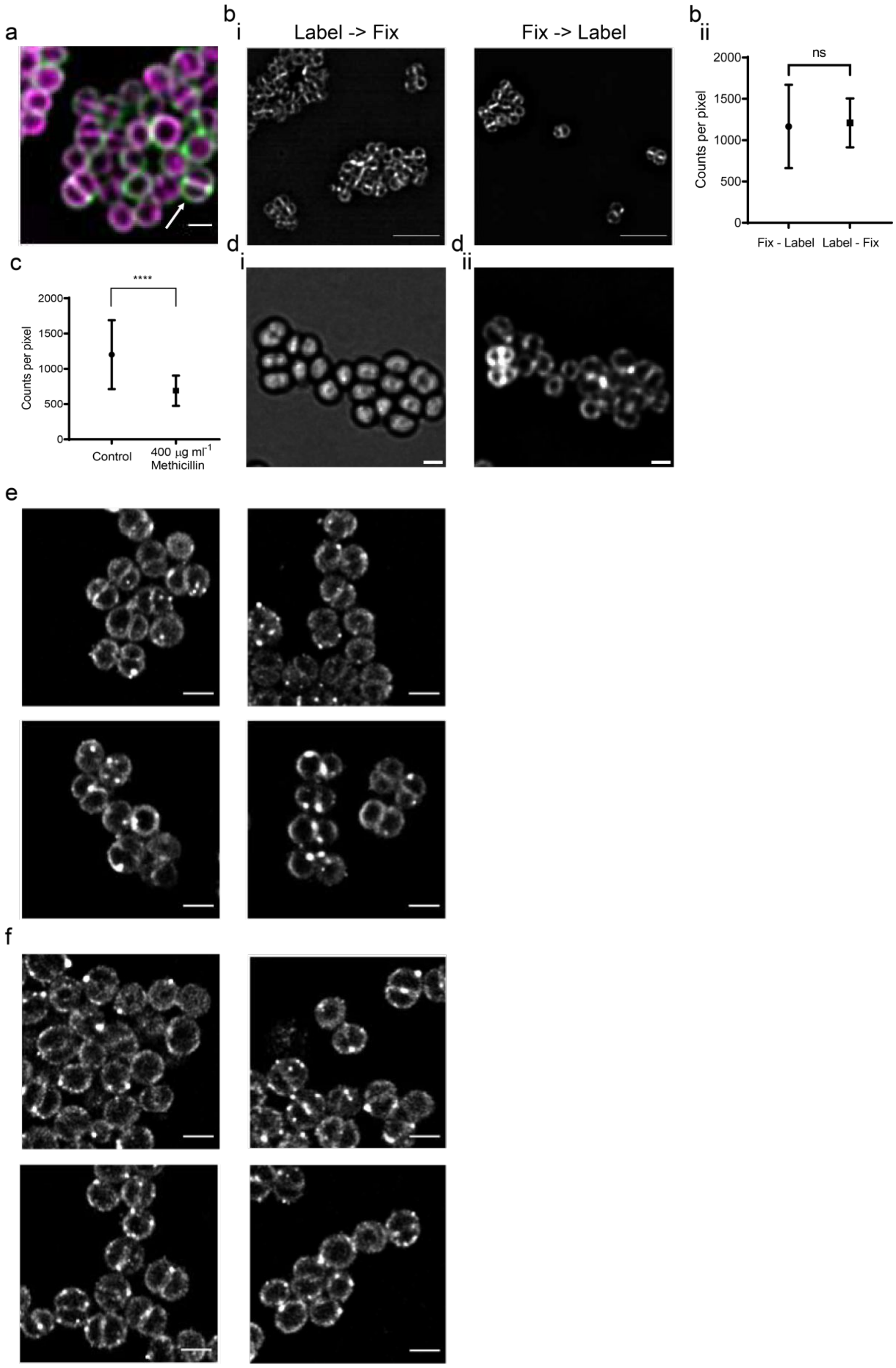

Figure S1: CephCy5 labels PBPs in *S. aureus.* Labelling with CephCy5. a) *S. aureus* SH1000 dual labelled with CephCy5 (localisation of PBPs, magenta) and NHS Ester- Atto488 (cell wall, green). Overlapping label is shown as white. Scale bar = 1 µm. bi) *S. aureus* SH1000 labelled with CephCy5

before and after fixing with paraformaldehyde. Scale bars = 5 µm. bii) Counts per pixel comparing CephCy5 labelling of *S. aureus* SH1000 pre and post fixation. Data analysed using an unpaired two-tailed t-test ($p = 0.5884$). c) Counts per pixel of labelling for *S. aureus* SH1000 labelled with CephCy5 in the presence of 400 µg ml^{-1} methicillin or absence (control). Data analysed using an unpaired two-tailed t-test ($p < 0.0001$). d) Representative *S. aureus* SH1000 labelled with CephCy5 imaged by deconvolution widefield microscopy. di) DIC image, dii) corresponding CephCy5. e) Representative dSTORM images of *S. aureus* SH1000 labelled with CephCy5. Scale bar = 1 µm. f) Representative dSTORM images of *S. aureus* SH4424 (*pbp3 pbp4*) labelled with CephCy5. Scale bar = 1 µm.

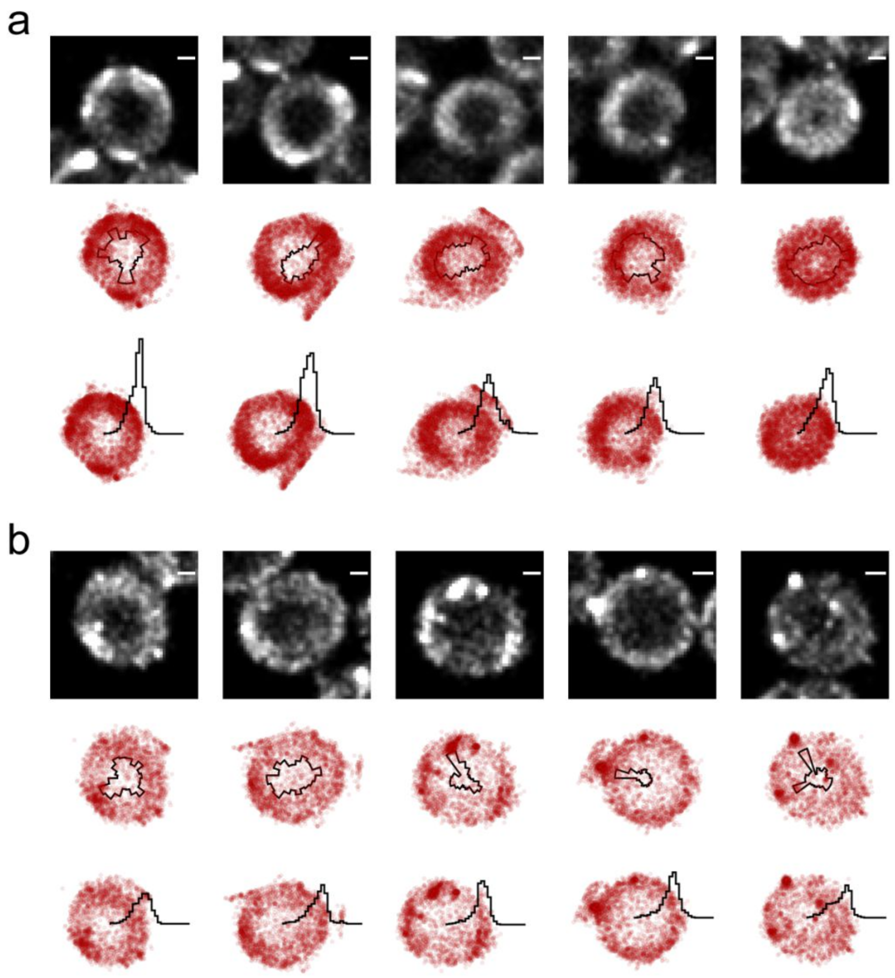

Figure S2: Analysis of CephCy5 molecular localisations. Analysis of CephCy5 localisations associated with septa, that lie in the plane of focus. Each panel shows the dSTORM image of an analysed septum (scale bar = 500 nm), below which are the localisations fitted to a circle and quantified due to their positioning around the septa, i.e., the angle with respect to an arbitrary 0° and their distance from the predicted centre of the septal annulus. a) SH1000 b) SH4424 (*pbp3 pbp4*).

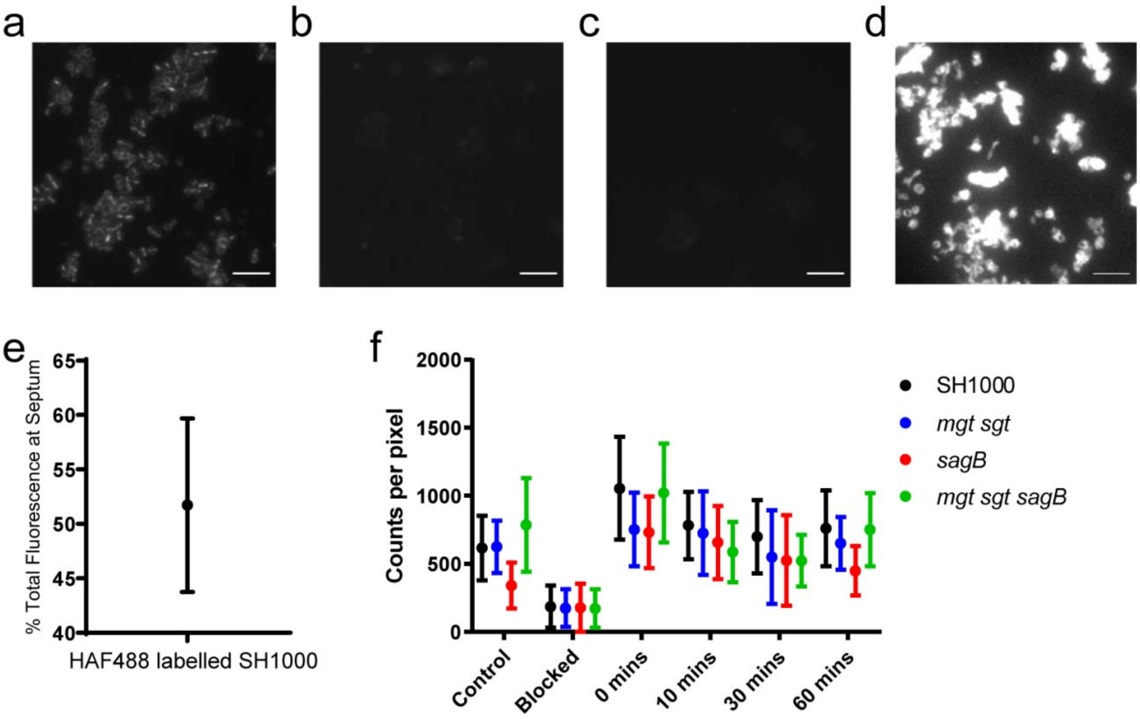

Figure S3: HAF488 labelling of reducing termini. a) *S. aureus* SH1000 labelled with HAF488. b) *S. aureus* SH1000 labelled with HAF488 following sodium borohydride treatment. c) *S. aureus* SH1000 labelled with HAF488 following hydroxylamine treatment. d) Extracted and purified *S. aureus* SH1000 sacculi labelled with HAF488. Scale bars = 5 µm. e) Percentage of total cell fluorescence found at the septum for SH1000 labelled with HAF488, n= 75. f) Analysis of brightness of HAF labelling following blocking with unlabelled HAF in SH1000, *Δmgt Δsgt*, *ΔsagB*, and Δ*mgt Δsgt ΔsagB* n=~200 for each condition.

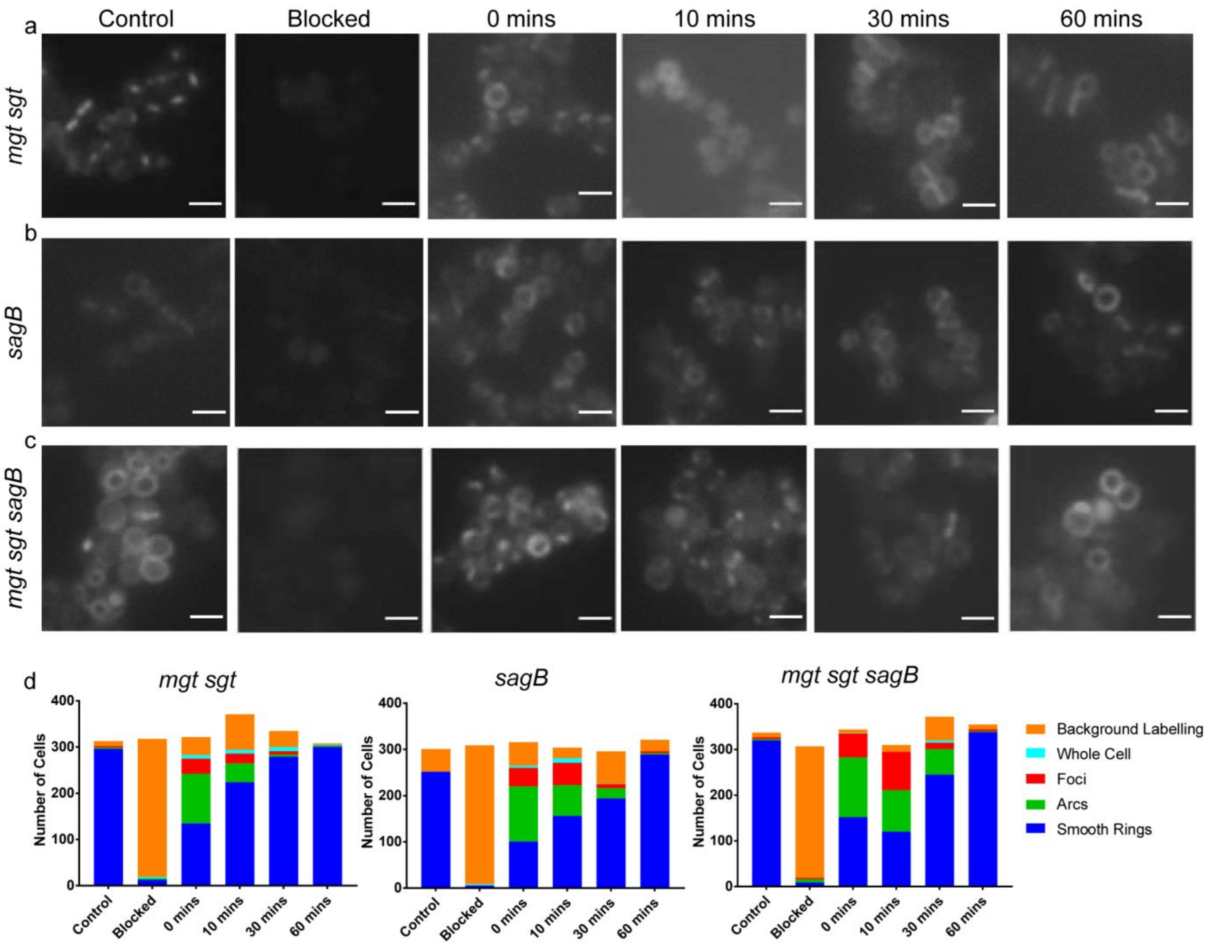

Figure S4: Role of cell wall homeostasis components in molecular localisation of reducing termini. Time course of the appearance of reducing termini (labelled with HAF488) following blocking with hydroxylamine as measured by fluorescence microscopy (a-c). a, *S. aureus* SH4644 *mgt sgtA*; b, *S. aureus* SH4608 *sagB*; c, *S. aureus* SH4659 *mgt sgtA sagB.* Scale bars = 1 µm. d, Analysis of observed HAF488 labelling pattern in cells from a-c. n=~300 for each sample. For examples of the designated features see Figure 2.

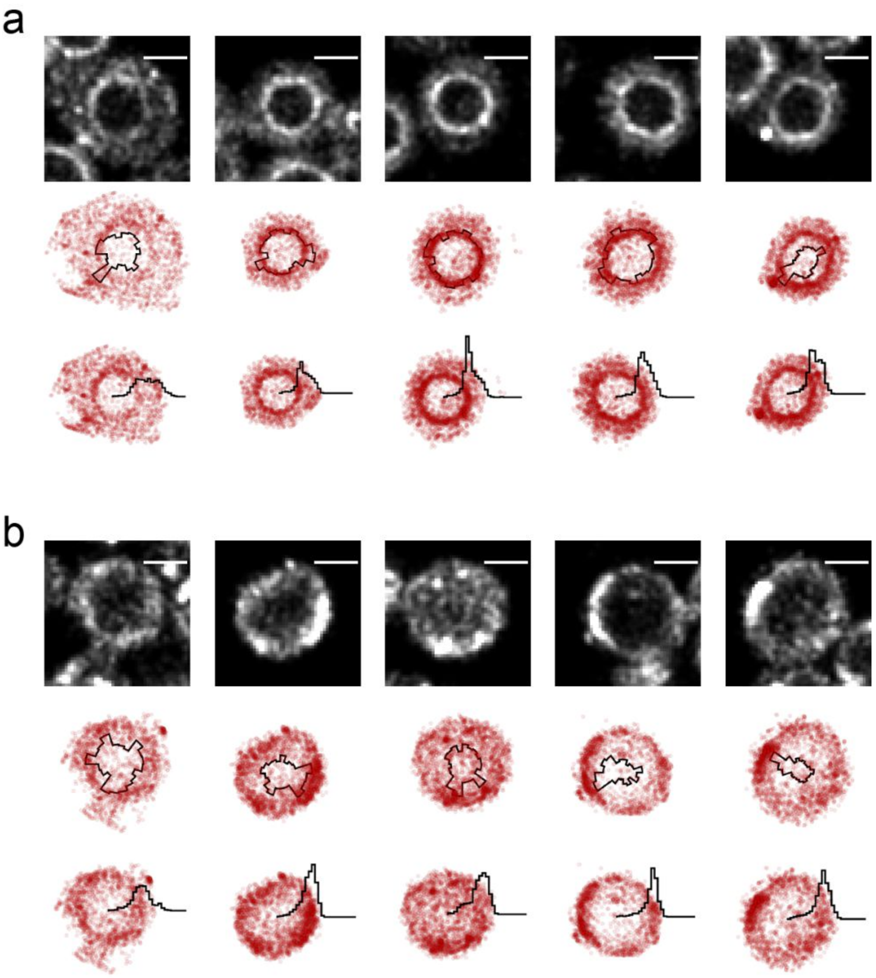

Figure S5: Analysis of AzTEG 647 molecular localisations. Analysis of AzTEG 647 localisations associated with septa, that lie in the plane of focus. Each panel shows the dSTORM image of an analysed septum (scale bar = 500 nm), below which are the localisations fitted to a circle and quantified due to their positioning around the septa, i.e., the angle with respect to an arbitrary 0° and their distance from the predicted centre of the septal annulus. a, *S. aureus* SH1000 labelled with AzTEG 647; b, *S. aureus* SH1000 treated with hydroxylamine and recovered for 10 min before AzTEG 647 labelling.

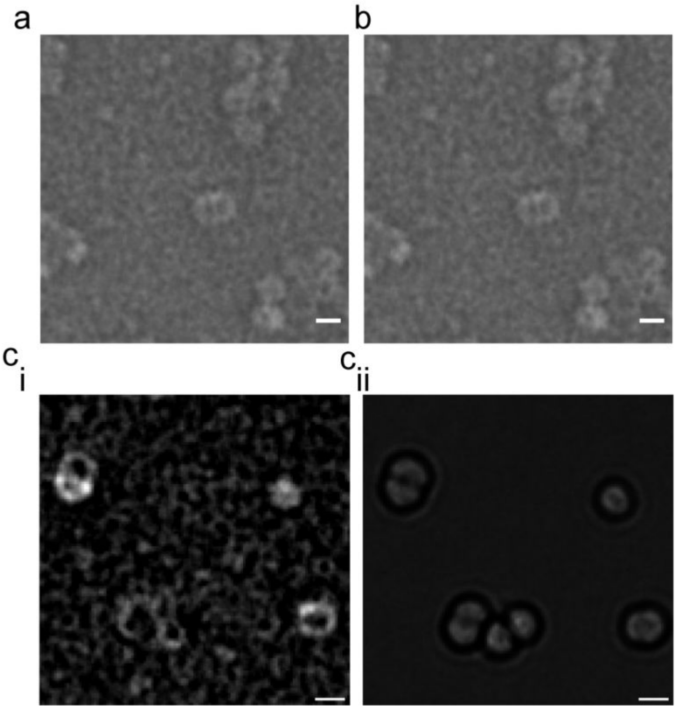

Figure S6: Labelling glycan strand synthesis using AzNAM. a-c) SH5097 (S. *aureus* SH1000 *geh:: P_{pcn}-amgK-murU*) was labelled for 15s with AzNAM clicked to Alexafluor647 and visualised by deconvolution microscopy. Scale bar = 1 µm. ci) Visualised AzNAM clicked to Alexafluor647 with corresponding DIC image (cii).

Materials & Methods

Bacterial Growth Conditions – Strains used in this study are listed in Table 1 and Plasmids are listed in Table 2. *S. aureus* was grown in Tryptone Soy Broth (TSB) at 37°C with aeration at 250rpm. For solid media 1.5% (w/v) Agar was added. Where antibiotics were required they were used at the following concentrations: erythromycin (5 µg ml^{-1}), lincomycin (25 µg ml^{-1}), spectinomycin (250 µg ml^{-1}), kanamycin (50µg ml^{-1}) and tetracycline (5 µg ml^{-1}, pKASBAR based strains; 0.5 ml^{-1}, pTET based strains).

Determination of minimum inhibitory concentration (MIC). MIC was determined in TSB and a serial dilution of the compound at different concentrations. The media was inoculated with *S. aureus* to an OD_{600} of 0.05 and left overnight at 37°C with aeration at 250rpm. The MIC was determined to be the lowest concentration at which there was no bacterial growth. Tubes containing TSB media and TSB media with culture were used control for each experiment. MICs were performed in triplicate.

Construction of Mutants - All mutants were confirmed by PCR & DNA sequencing.

PBP Mutants. NE420 (*S. aureus* JE2 *pbp3::Tn*) was obtained from the NARSA Library [3]. SH1000 was transduced with a phage lysate from NE420 resulting in SH4421. To swap the erythromycin resistance cassette to a spectinomycin cassette SH4421 was transduced with a phage lysate from NE3003 (*S. aureus* RN4220 pSPC). Transformants were selected at 28°C, then incubated at 42°C to initiate the single crossover event. Resulting recombinants were grown and passaged 4 times at 28°C to allow double crossover resulting in SH4422 *(S. aureus* SH1000 *pbp3::spec)*. SH4422 was subsequently transduced with a phage lysate from SH4425 *(S. aureus* SH1000 *pbp4::Tn*) resulting in SH4424 (*S. aureus* SH1000 *pbp3::spec pbp4::Tn*).

Monofunctional Transglycosylase Mutants. NE596 (*S. aureus* JE2 *mgt::Tn*) and NE267 (*S. aureus* JE2 *sgtA::Tn*) were obtained the NARSA Library [3]. SH1000 was transduced with phage lysates from NARSA strains resulting in SH4628 & SH4629 respectively. The erythromycin cassette in SH4629 was swapped to a tetracycline cassette in the same manner as in SH4422 with NE3005 (*S. aureus* RN4220 pTET) as the donor strain resulting in SH4643 (*S. aureus* SH1000 *sgtA::tet*). A phage lysate from SH4628 was transduced into SH4643 to produce the double mutant SH4644 (*S. aureus* SH1000 *mgt::Tn sgtA::tet*). SH4644 was subsequently transformed with a phage lysate from SH4608 (*S. aureus* SH1000 *sagB::kan*), resulting in SH4659 *S. aureus* SH1000 (*mgt::Tn sgtA::tet sagB::kan*).

AmgK MurU Producing Strains. To create a *S. aureus* strain expressing AmgK & MurU under the control of P_{pcn} a codon optimised gene fragment was synthesised with the final amino acid of AmgK

overlapping with the start codon of MurU (Invitrogen). The gene construct was amplified by PCR and inserted into pKASBAR [7] and the resulting plasmid pKASBAR-P_{pcn} *amgK murU* was electroporated into CYL316 and integration into the *geh* locus was confirmed by disruption of lipase production on Baird-Parker medium (SH4969). The chromosomal region containing the plasmid was then transduced into SH1000 producing SH5097 (*S. aureus* SH1000 *geh::* P_{pcn}*-amgK-murU*).

Sequence of Ppcn AmgK MurU. The sequence of Ppcn AmgK MurU inserted into pKASBAR is reported below. Ppcn is coloured red, AmgK blue, MurU green.

GAAACGAGGTCATCATTTCCTTCCGAAAAAACGGTTGCATTTAAATCTTACATATGTAATACTTTCAAAGACTA
CATTTGTAAGATTTGATGTTTGAGTCGGCTGAAAGATCGTACGTACCAATTATTGTTTCGTGATTGTTCAAGCC
ATAACACTGTAGGGATAGTGGAAAGAGTGCTTCATCTGGTTACGATCAATCAAATATTCAAACGGAGGGAGA
CGATTTTGATGCCAGAGCACGACGTTCGATTACAACAGTTGACAGTATGGTTAGATGAACAATTGAATGATTT
ATTCAGAGATAACGCGTGGGGAGAAGTGCCTGCGGGCAGTTTGACGGCTGCATCAAGTGACGCTTCATTCCG
AAGATATTTCCGTTGGCAGGGCGCAGGTCACTCATTTGTAATTATGGATGCACCACCTCCACAAGAGAATTGT
CGACCTTTTGTGGCTATTGACCACTTATTGGCAAGTGCAGACGTTCACGTTCCGTTGATCCACGCTCAAGACTT
GGAAAGAGGCTTCTTATTGTTGGGAGATTTAGGCACTCAGACTTACTTGGATATTATAAATGCGGATAACGCG
GATGGTTTATTTGCGGACGCAATAGATGCGTTATTGAAGTTTCAGCGTTTACCGATGGATGCTCCTTTACCATC
TTACGATGATGCTTTGTTGCGACGTGAAGTAGAATTATTCCCGGAGTGGTACGTAGGTCGTGAATTAGGCTTA
ACTTTCACAGATGCACAAAAGGCGACATGGCAACGTGTCTCTCAATTGTTAATAGATAGTGCATTGGCTCAGC
CAAAAGTGTTAGTTCACCGAGATTACATGCCACGAAACTTGATGCAGAGTACTCCAAATCCGGGTGTATTAGA
CTTTCAGGACGCGGTCTATGGCCCAGTAACATACGACATCACTTGTTTGTTCAAAGACGCTTTCGTTTCTTGGC
CTCAAGCTAGAGTTGAGGGTTGGTTGGGTGATTACTGGCAACAAGCGCAGGCTGCAGGCATTCCAGTGCATG
CAGAGTTTGAGGCTTTTCATCGAGCATCAGACTTGATGGGTGTTCAGCGTCACTTAAAGGTAATTGGCATTTTT
GCACGTATTTGCCATCGTGATGGCAAACCTAGATACTTAGGCGATGTTCCACGATTCTTCGCTTATATAAACGA
GGTAATAGGTCGTAGACCGGAATTAGCTGAGTTAGGTGAGTTGATCGCTGAATTACAGGCTGGAGCGCGTGC
ATGAAAGCAATGATATTGGCGGCAGGAAAAGGAGAACGAATGCGTCCTTTGACGTTGCACACACCTAAACCG
TTAGTGCCGGTTGCAGGACAGCCTTTAATAGAATATCATTTACGAGCGTTGGCGGCTGCGGGTGTCACGGAG
GTAGTAATAAACCATGCGTGGTTGGGACAACAGATTGAGGACCATTTGGGTGATGGCTCAAGATTCGGTTTG
TCAATCCGTTATTCACCAGAGGGCGAGCCATTGGAGACAGGTGGCGGTATCTTTAAAGCGTTACCGTTATTGG
GCGATGCGCCTTTTTTGTTAGTAAATGGAGACGTTTGGACTGATTACGACTTTGCTAGATTGCAAGCGCCATTG
CAAGGCTTAGCTCATTTAGTTTTGGTAGACAACCCTGGTCATCACGGTCGTGGCGATTTCAGATTAGTAGGAG
AGCAAGTTGTAGACGGAGATGACGCGCCGGGAACATTAACGTTTTCTGGCATTAGTGTGTTGCACCCTGCATT
GTTTGAGGGTTGCCAGGCTGGTGCATTTAAGTTAGCACCGTTGTTAAGACAAGCGATGGCAGCTGGTAAAGT
TAGTGGTGAACACTACCGTGGACACTGGGTGGACGTTGGTACGTTAGAACGATTGGCGGAAGCAGAGAGTTT
AATTGGTGAACGTGCATGA

***In vitro* labelling of *S. aureus* PBPs with fluorescent β-lactams.** The membrane fraction of *S. aureus* was prepared as previously described [7] with the following modifications. Cells were grown to an OD_{600}~1, recovered by centrifugation (5,000 x g, 10 min), washed three times by resuspension and centrifugation (5,000 x g, 10 min, 4°C) in PBS. Cells were resuspended in TBSI (50 mM Tris, 100 mM NaCl, pH 8, plus Complete Protease Inhibitor Cocktail, Roche) to an OD_{600} of 300 and broken using 0.1 mm silica spheres (Lysing Matrix B) and FastPrep Homogenizer (MP Biomedicals) in 12 cycles of 30 s, at maximum speed (6.5 m s^{-1}), with 5 min incubation on ice between cycles. Cell lysates were centrifuged (5,000 x g, 10 min, 4°C) to remove unbroken cells. The supernatant was then spun twice (15,000 x g, 10 min, 4°C) to sediment cell wall material. The membrane fraction was recovered from the supernatant by centrifugation (70,000 x g, 60 min, 4°C) and the pellet (membranes) was resuspended in PBS. The total protein concentration was estimated by Bradford assay. Membrane proteome sample (25 µg; 20 µl of 1.3 mg ml^{-1}) was incubated with 25 µM BocillinFL or 10, 20, 30 or 40 µM CephCy5 for 10 min at 37°C. The reaction was stopped by addition of 5x SDS-PAGE loading buffer and heating for 10 min at 90°C. The samples were run on a 10% SDS-PAGE gel and visualized using a BioRad ChemiDoc MP Imaging system.

Labelling with CephCy5. *S. aureus* was grown to mid exponential phase and 1 ml samples were taken. For live cell labelling CephCy5 was added at 20 µM and cells were labelled at 37°C for 5 mins, cells were the collected by centrifugation and fixed with 4% w/v paraformaldehyde. For fixed cell labelling 1ml samples were fixed using 4% w/v paraformaldehyde then collected using centrifugation. The cells were resuspended in 500 µl PBS containing CephCy5 at 20 µM and labelled for 5 mins.

Labelling with HAF488 and AzTEG 647. 1ml samples of *S. aureus* were collected by centrifugation at resuspended in PBS containing 50 µg ml^{-1} HAF488 and incubated at room temperature for 5 minutes with rotation. Cells were then collected by centrifugation and fixed. For samples labelled with AzTEG 647, the alkyne dye was attached using copper catalysed H reaction using the Click-iT Cell reaction Buffer kit from ThermoFisher, using manufacturers protocols with dyes at 5 µg ml^{-1}. Blocking of samples was carried out with cells collected by centrifugation and resuspended with PBS containing 2.5% (w/v) Hydroxylamine and incubated at 37°C for 10 minutes, cells were washed once in PBS before labelling or resuspension in TSB.

Labelling with AzNAM. SH5097 (*S. aureus* SH1000 *geh:: P_{pcn}-amgK-murU*) were grown to mid-exponential phase. 1ml cells were collected by centrifugation and resuspended in 200 µl TSB containing 2 mg ml^{-1} AzNAM. AzNAM was produced as previously described[19, 20]. Cells were incubated at 37°C for required time (5 mins) depending on requirements. Cells were then collected and fixed using 4% w/v paraformaldehyde. The alkyne dye was attached using copper catalysed click reaction using the Click-iT Cell reaction Buffer kit from ThermoFisher using manufacturers protocols with dyes at 5 µg ml^{-1}.

OMX Microscopy. Coverslips (High-precision, No.1.5H, 22 × 22 mm, 170 ± 5 µm, Marienfeld) were sonicated for 15 min in 1 M KOH, washed with water and incubated in poly-l-lysine solution for 30 min. Coverslips were then further washed and dried with nitrogen. Fixed cells were then dried onto the coverslips with nitrogen and mounted on slides with ~5 µl Slow Fade Diamond (Invitrogen).

Widefield deconvolution and Structured Illumination Microscopy were carried out using a v4 DeltaVision OMX 3D-SIM system fitted with a Blaze module (Applied Precision, GE Healthcare, Issaquah, USA). For widefield imaging, samples were illuminated using a 6 colour solid-state illuminator (LED). The objective was a 60 x 1.42 oil plan apochromat lens, the system has a standard BGR filter set and uses scientific CMOS cameras. For 3D-SIM, samples were illuminated using laser illumination. For each z slice, samples were imaged in 5 phase shifts and 3 angles, z-steps were 0.125 nm. Reconstructions were performed with the Softworx software (GE Healthcare) using OTFs optimised for the specific wavelength and oil used. The same software was used for deconvolution.

STORM. Localisation microscopy was carried out using a Nikon Ti-NS N-STORM version 1 in continuous mode. The objective used was an SR Apo TIRF 100x NA 1.49 and images detected using EMCCD camera (Andor DU-897) using the 17 MHz 16-bit mode with an EM Multiplier Gain of 300 and a conversion gain of 3. AlexaFluor647 & CephCy5 were both imaged using the OBIS 647 laser and the NSTORM cube. Imaging was done under oblique illumination but not full TIRF and 405nm laser excitation was applied when required.

Images were reconstructed using the Nikon Elements software, with drift correction applied via cross-correlation of localisations in ThunderSTORM, an ImageJ/Fiji plugin [8].

Analysis of localisation microscopy data. Localisations from septa lying in the plane of focus were manually selected from fields. The localisations in these rings were then fitted to a circle of best fit[4].

Synthetic Methods and Characterisation

All reactions conducted with anhydrous solvents were performed under a nitrogen atmosphere using acetone-washed, flame-dried glassware with magnetic stirring and if required heated through the use of Dry Syn^{TM} blocks. All chemicals were purchased from commercial suppliers and used as received without further purification. Reactions that were performed at 0 °C and –78 °C used water/ice baths and acetone/dry ice baths, respectively.

Analytical thin layer chromatography (TLC) was carried out utilising aluminium-backed Merck TLC plates (silica gel 60 F254) and visualised with UV light (254 nm), basic $KMnO_4$ or ninhydrin solution. Flash column chromatography was performed using VWR International Silica Gel 40-63µ 60Å as the stationary phase. Columns were typically packed as a slurry and equilibrated with the appropriate solvent system prior to use.

^{1}H and ^{13}C NMR spectra were recorded on either Bruker AV 400, AVIIIHD 400 or AVIIIHD 500 spectrometers at 298K. Chemical shifts for ^{1}H NMR spectra are reported as δ in units of parts per million (ppm) downfield from $SiMe_4$ (δ 0.0) and relative to the signal of $CHCl_3$-d (δ 7.26, singlet). Coupling constants (J) are given in Hertz (Hz) to the nearest 0.5 Hz and were corrected. ^{13}C NMR spectra are recorded using the JMOD or DEPT method and reported as δ in ppm downfield from $SiMe_4$ (δ 0.0) and relative to the signal of chloroform-d (77.0, triplet). Mass spectra (m/z) were recorded on a 'VG'-Autospec for Electron Ionisation (EI) or a 'Waters'-LCT for Electrospray (ES). Infrared spectra were recorded on a Perkin-Elmer 1600 FT-IR using a Universal diamond ATR top-plate. Specific rotations were performed at 298K on an Optical Activity Ltd. AA-10 automatic polarimeter at 589 nm (Na D-line) and $[\alpha]_D$ values are given in 10^{-1} deg cm^2 g^{-1}. HPLC purification was carried out on a Varian OD analytical system using a XBridge C_{18} column (19 mm × 250 mm) with flow rate of 1.00 mL/min. All compounds purified by HPLC were assumed to be salts of the buffer used. LC-MS analysis was carried out on a Waters Alliance 2696 HPLC system using a Phenomenex Kinetex 2.6µm 100Å C_{18} column (50 mm x 2.10 mm) and a flow rate of 0.4 mL/min with a gradient of 5 – 95% MeCN: H_2O (0.1% TFA) over 30 minutes.

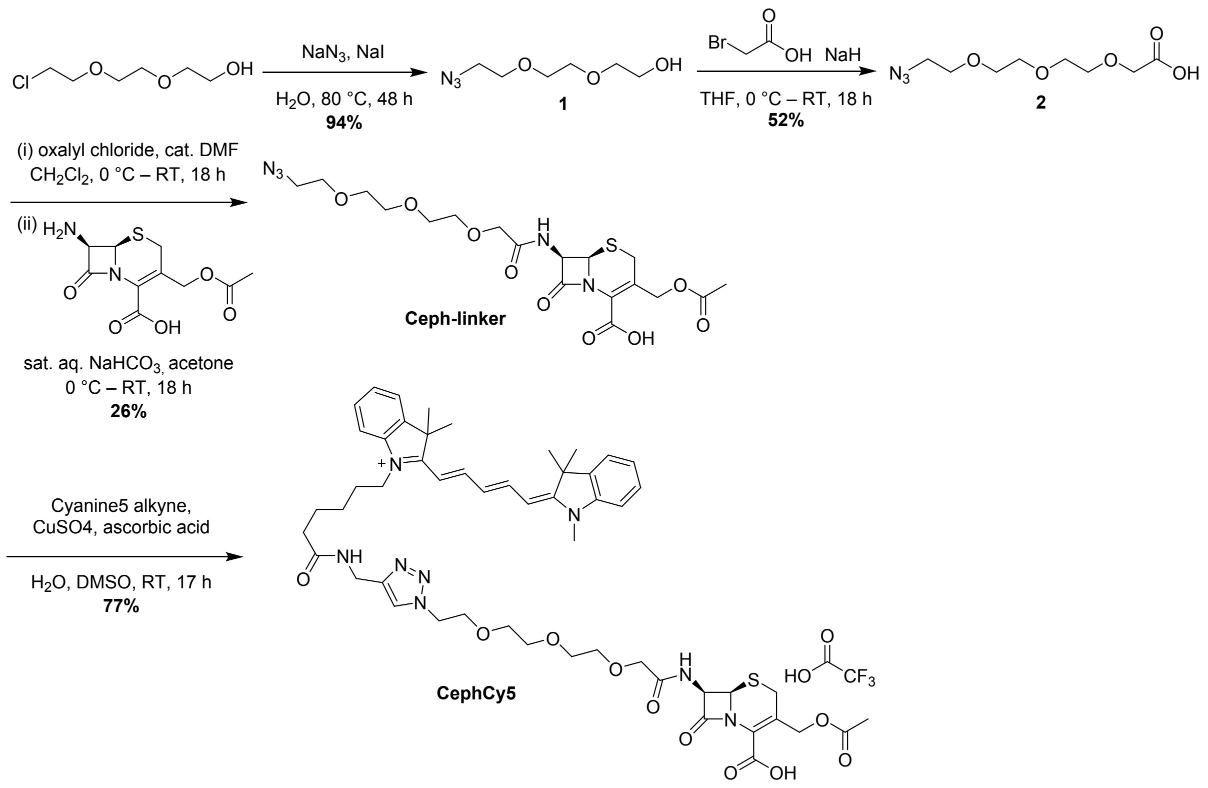

Scheme 1: Synthesis of CephCy5

2-(2-(2-Azidoethoxy)ethoxy)ethan-1-ol (1)[9]: A solution of 2-[2-(2-chloroethoxy)ethoxy]ethanol (1.72 mL, 11.9 mmol), sodium azide (1.55 g, 23.8 mmol) and sodium iodide (15.9 mg, 2.3 mmol) in H_2O (40 mL) was stirred at 80 °C for 24 hours. The reaction was cooled to room temperature and additional sodium azide (775 mg, 11.9 mmol) and sodium iodide (15.9 mg, 2.3 mmol) were added. The solution was stirred at 80 °C for a further 24 hours, cooled to room temperature and extracted with CH_2Cl_2 (4 × 50 mL). The combined organic extracts were dried over $MgSO_4$, filtered and concentrated *in vacuo* to afford the title compound 2-(2-(2-azidoethoxy)ethoxy)ethan-1-ol as a clear oil which did not require further purification (1.93 g, 94%); δ_H ($CDCl_3$, 400 MHz): 3.80 – 3.72 (m, 2H), 3.66 – 3.72 (m 6H), 3.60 – 3.66 (m, 2H), 3.37 – 3.46 (m, 2H), 2.26 (br s, 1H); HRMS (ES^+) m/z: [M + Na^+] Calcd $C_6H_{13}N_3O_{35}Na$ 198.0866; Found 198.0855.

2-(2-(2-(2-Azidoethoxy)ethoxy)ethoxy)acetic acid (2)[10]: Sodium hydride (60% mineral oil, 192 mg, 22.8 mmol) in anhydrous THF (10 mL) was cooled to 0 °C and polyether 2-(2-(2-azidoethoxy)ethoxy)ethan-1-ol (1.00 g, 5.71 mmol) was added. This was stirred for 15 minutes at 0 °C prior to adding bromoacetic acid (1.19 g, 8.57 mmol) and stirring the mixture at room temperature for 18 hours. The reaction mixture was quenched with MeOH (10 mL), concentrated *in vacuo* and the residue partitioned between CH_2Cl_2 (50 mL) and 1M HCl (30 mL). The organic layer was separated, washed with brine (30 mL), dried over $MgSO_4$, filtered and concentrated *in vacuo*. Purification by dry flash column chromatography using silica gel and an eluent of 0 – 100% ethyl

acetate: petroleum ether 40 – 60 afforded the title compound **2** as a clear oil (700 mg, 52%); δ_H (400 MHz, $CDCl_3$): 4.17 (s, 2H), 3.79 – 3.75 (m, 2H), 3.74 – 3.70 (m, 4H), 3.70 – 3.65 (m, 4H), 3.44 – 3.37 (m, 2H); HRMS (ES^+) m/z: [M + Na^+] Calcd for $C_8H_{15}N_3O_5Na$ 256.0909; Found 256.0900.

(6*R*,7*R*)-3-(Acetoxymethyl)-7-(2-(2-(2-(2-azidoethoxy)ethoxy)ethoxy)acetamido)-8-oxo-5-thia-1-azabicyclo[4.2.0]oct-2-ene-2-carboxylic acid (Ceph-linker): A solution of 2-(2-(2-(2-azidoethoxy)ethoxy)ethoxy)acetic acid (128 mg, 0.550 mmol) in anhydrous CH_2Cl_2 (4 mL) was cooled to 0 °C. Oxalyl chloride (50 µL, 0.590 mmol) and DMF (4 drops) were added and the solution stirred at room temperature for 18 hours. The mixture was concentrated *in vacuo*, dissolved in acetone (0.75 mL) and added to a suspension of 7-aminocephalosporanic acid (100 mg, 0.370 mmol) in sat. aq. $NaHCO_3$ (2.5 mL) and acetone (0.75 mL) at 0 ˚C. The resulting mixture was stirred at room temperature for 18 hours. The mixture was acidified to pH 2 with 1 M HCl and extracted with CH_2Cl_2 (3 × 10 mL). The combined organic extracts were dried over Na_2SO_4, filtered and concentrated *in vacuo*. Purification by preparative HPLC using an eluent of 30% MeCN: H_2O afforded the title compound **Ceph-linker** as a pale yellow oil (46 mg, 26%); $[\alpha]_D^{26}$ + 52.2 (*c* 0.9 in $CHCl_3$); υ_{max} (ATR) / cm^{-1} 3304, 2915, 2879, 2103, 1779, 1731, 1680; δ_H ($CDCl_3$, 400 MHz): 7.80 (d, 1H, *J* = 9.0 Hz), 6.93 (br s, 1H), 5.84 (dd, 1H, *J* = 9.0 Hz, 5.0 Hz), 5.11 (d, 1H, *J* = 13.5 Hz), 5.02 (d, 1H, *J* = 5.0 Hz), 4.90 (d, 1H, *J* = 13.5 Hz), 4.10 (ABq, 2H, J_{A-B} = 16.0 Hz), 3.61 – 3.75 (m, 10H), 3.58 (d, 1H, *J* = 19.0 Hz), 3.41 (d, 1H, *J* = 19.0 Hz), 3.33 – 3.38 (m, 2H), 2.08 (s, 3H); δ_C ($CDCl_3$, 101 MHz): 171.4, 171.0, 164.8, 163.2, 126.9, 125.8, 71.4, 70.8, 70.7, 70.5, 70.3, 70.2, 63.3, 58.8, 57.5, 50.8, 26.6, 20.9; HRMS (ESI^+) m/z: [M + Na^+] Calcd for $C_{18}H_{25}N_5O_9SNa$ 510.1265; Found 510.1271.

1-[5-({[1-(2-{2-[2-({[(6*R*,7*R*)-3-[(Acetyloxy)methyl]-2-carboxy-8-oxo-5-thia-1-azabicyclo[4.2.0]oct-2-en-7-yl]carbamoyl}methoxy)ethoxy]ethoxy}ethyl)-1H-1,2,3-triazol-4-yl]methyl}carbamoyl)pentyl]-3,3-dimethyl-2-[(1*E*,3*E*)-5-[(2*E*)-1,3,3-trimethyl-2,3-dihydro-1H-indol-2-ylidene]penta-1,3-dien-1-yl]-3H-indol-1-ium; trifluoroacetic acid (CephCy5): Ascorbic acid (0.510 mg, 0.00288 mmol) in H_2O (20 µL) was added to a solution of (6*R*,7*R*)-3-(acetoxymethyl)-7-(2-(2-(2-(2-azidoethoxy)ethoxy)ethoxy)acetamido)-8-oxo-5-thia-1-azabicyclo[4.2.0]oct-2-ene-2-carboxylic acid (0.92 mg, 0.00180 mmol) and cyanine5 alkyne (1.00 mg, 0.00180 mmol) in DMSO (200 µL). This was stirred for 2 minutes and then $CuSO_4$ (0.230 mg, 0.00144 mmol) in H_2O (20 µL) was added and the reaction mixture stirred for 17 hours. The reaction mixture was then purified directly by preparative HPLC using an eluent of 50% MeCN: H_2O (0.1% TFA) to afford the title compound **CephCy5** as a blue solid (1.4 mg, 77%); λ_{max}(MeCN)/nm 641 (ε/dm^3 mol^{-1} cm^{-1} 11 686); HRMS (ESI^+) m/z: [M + H^+] Calcd for $C_{53}H_{67}N_8O_{10}S$ 1007.4695; Found 1007.4681; Fig. S7 shows LC-MS data demonstrating purity below (Channel 1 = UV_{650}). N.B. Sample showed contaminants present; these were illustrated to have been from the LC-MS column itself using a blank background run, and not the sample.

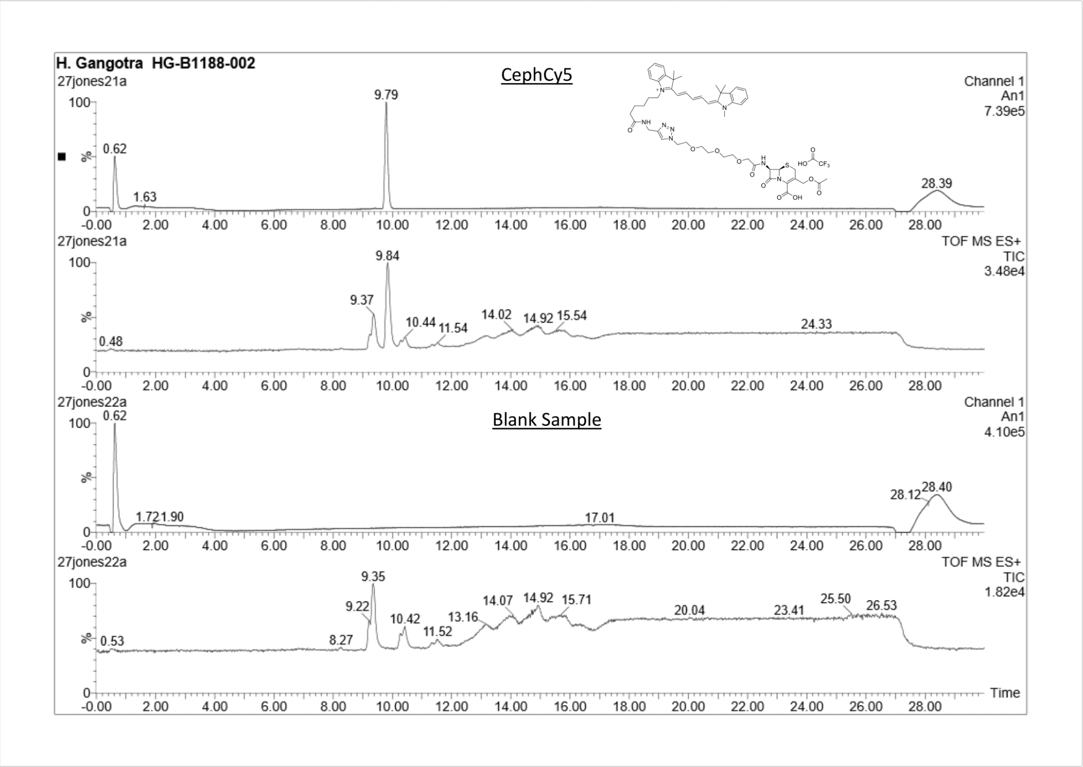

Fig. S7 LC-MS of CephCy5 and blank control

Preparation of AzTEG 647

15 µL of Alexafluor 647 alkyne dye was added to the required concentrations of 1-[2-(aminooxy)ethoxy]-2-(2-azidoethoxy)ethane [11] in 220 µL of click buffer, followed by addition of 15 µL of $CuSO_4$ and 25 µL of additive at RT. Biological samples were incubated with the above resulting mixture according to experimental conditions.

NMR Spectra

Compound 1.1.fid
HG-A1131-001
PROTON.s CDCl3 {C:\NMRData\Jones_current_year} chp16h 27

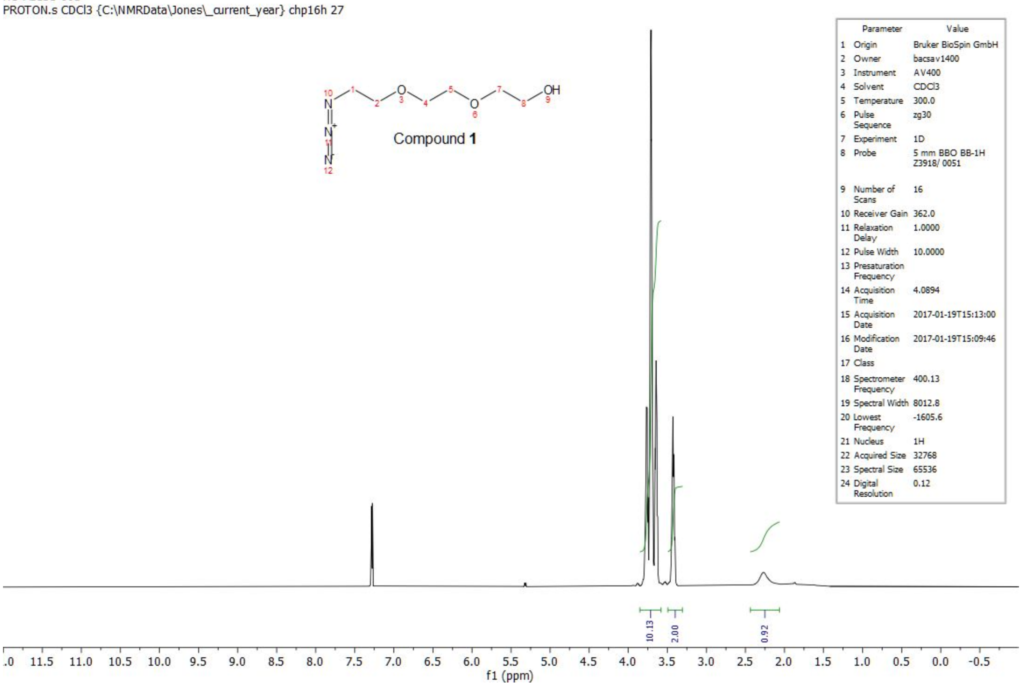

Magnus_2017_01_23.2.fid
HG-A1133-002
PRO CDCl3 {C:\NMRData\Jones_current_year} chp16h 48

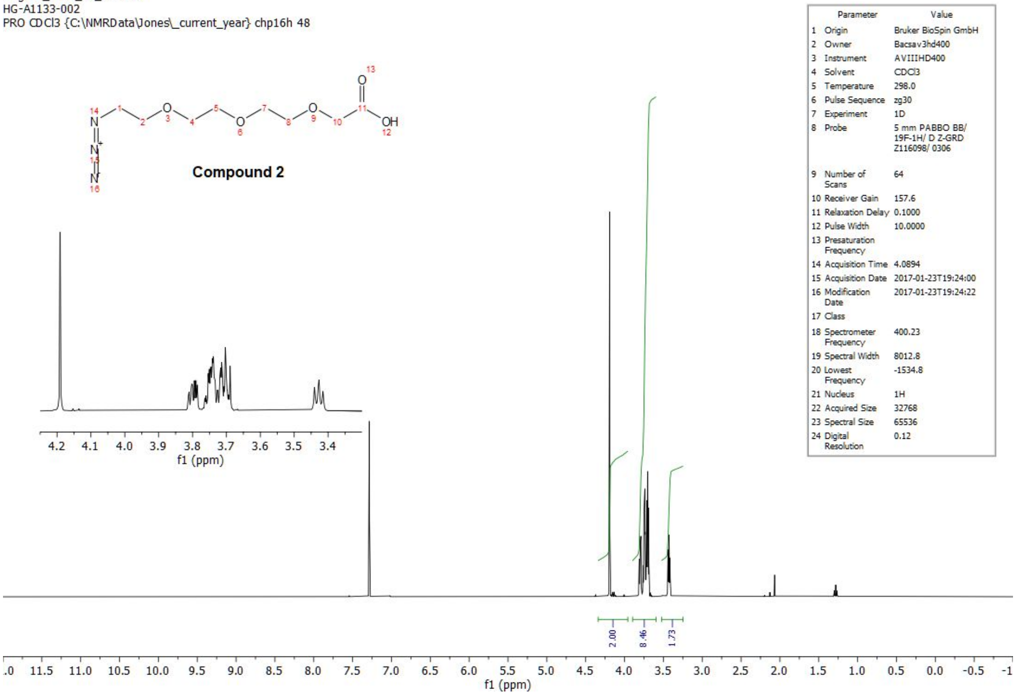

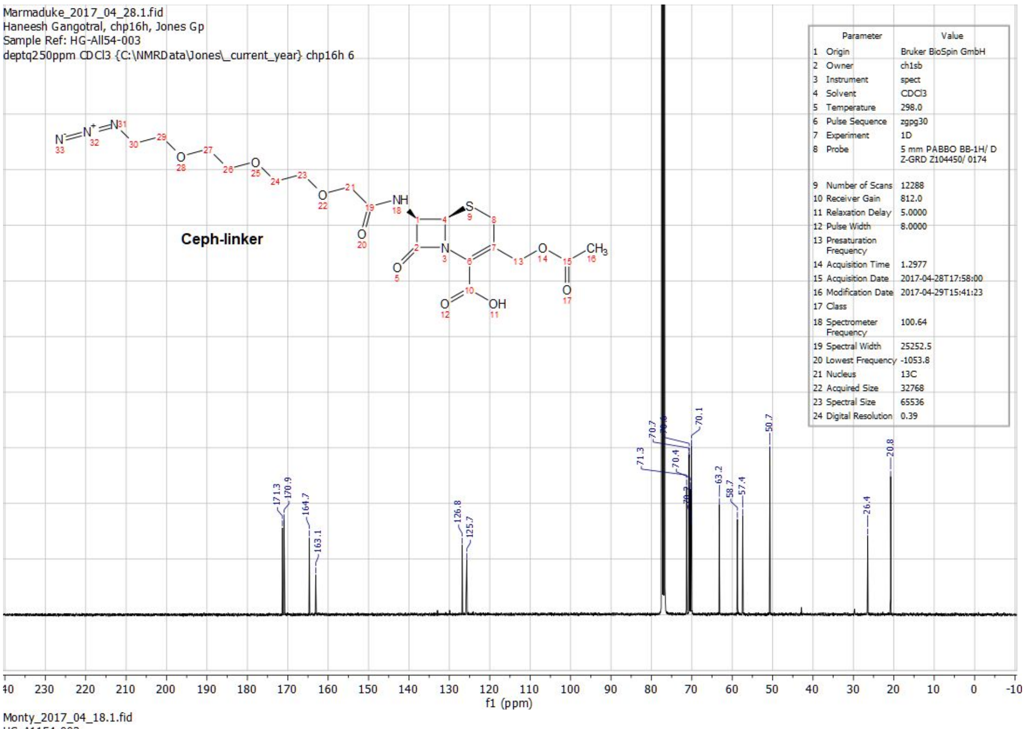

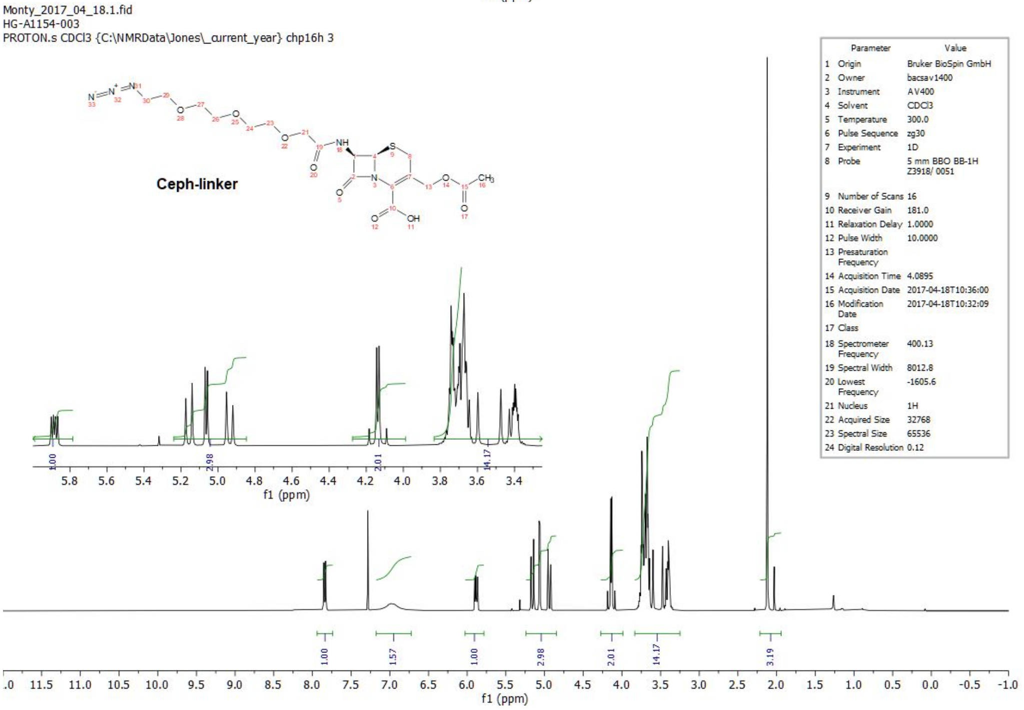

Fig. S8: NMR spectra of compounds from Scheme 1, synthesis of CephCy5

References

(1) Horsburgh, M. J.; Aish, J. L.; White, I. J.; Shaw, L.; Lithgow, J. K.; Foster, S. J. SigmaB Modulates Virulence Determinant Expression and Stress Resistance: Characterization of a Functional RsbU Strain Derived from Staphylococcus Aureus 8325-4. *J. Bacteriol.* **2002**, *184* (19), 5457–5467. https://doi.org/10.1128/JB.184.19.5457-5467.2002.

(2) Lee, C. Y.; Buranen, S. L.; Ye, Z. H. Construction of Single-Copy Integration Vectors for Staphylococcus Aureus. *Gene* **1991**, *103* (1), 101–105. https://doi.org/10.1016/0378-1119(91)90399-v.

(3) Fey, P. D.; Endres, J. L.; Yajjala, V. K.; Widhelm, T. J.; Boissy, R. J.; Bose, J. L.; Bayles, K. W. A Genetic Resource for Rapid and Comprehensive Phenotype Screening of Nonessential Staphylococcus Aureus Genes. *mBio* **2013**, *4* (1), e00537-00512. https://doi.org/10.1128/mBio.00537-12.

(4) Lund, V. A.; Wacnik, K.; Turner, R. D.; Cotterell, B. E.; Walther, C. G.; Fenn, S. J.; Grein, F.; Wollman, A. J.; Leake, M. C.; Olivier, N.; Cadby, A.; Mesnage, S.; Jones, S.; Foster, S. J. Molecular Coordination of Staphylococcus Aureus Cell Division. *eLife* **2018**, *7*, e32057. https://doi.org/10.7554/eLife.32057.

(5) Wheeler, R.; Turner, R. D.; Bailey, R. G.; Salamaga, B.; Mesnage, S.; Mohamad, S. A. S.; Hayhurst, E. J.; Horsburgh, M.; Hobbs, J. K.; Foster, S. J. Bacterial Cell Enlargement Requires Control of Cell Wall Stiffness Mediated by Peptidoglycan Hydrolases. *mBio* **2015**, *6* (4), e00660. https://doi.org/10.1128/mBio.00660-15.

(6) Sutton, J. A. F.; Carnell, O. T.; Lafage, L.; Gray, J.; Biboy, J.; Gibson, J. F.; Pollitt, E. J. G.; Tazoll, S. C.; Turnbull, W.; Hajdamowicz, N. H.; Salamaga, B.; Pidwill, G. R.; Condliffe, A. M.; Renshaw, S. A.; Vollmer, W.; Foster, S. J. Staphylococcus Aureus Cell Wall Structure and Dynamics during Host-Pathogen Interaction. *PLOS Pathog.* **2021**, *17* (3), e1009468. https://doi.org/10.1371/journal.ppat.1009468.

(7) Bottomley, A. L.; Kabli, A. F.; Hurd, A. F.; Turner, R. D.; Garcia-Lara, J.; Foster, S. J. Staphylococcus Aureus DivIB Is a Peptidoglycan-Binding Protein That Is Required for a Morphological Checkpoint in Cell Division. *Mol. Microbiol.* **2014**, *94* (5), 1041–1064. https://doi.org/10.1111/mmi.12813.

(8) Ovesný, M.; Křížek, P.; Borkovec, J.; Svindrych, Z.; Hagen, G. M. ThunderSTORM: A Comprehensive ImageJ Plug-in for PALM and STORM Data Analysis and Super-Resolution Imaging. *Bioinforma. Oxf. Engl.* **2014**, *30* (16), 2389–2390. https://doi.org/10.1093/bioinformatics/btu202.

(9) Shi, W.; Dolai, S.; Averick, S.; Fernando, S. S.; Saltos, J. A.; L'Amoreaux, W.; Banerjee, P.; Raja, K. A General Methodology toward Drug/Dye Incorporated Living Copolymer-Protein Hybrids: (NIRF Dye-Glucose) Copolymer - Avidin/BSA Conjugates as Prototypes. *Bioconjug. Chem.* **2009**, *20* (8), 1595–1601. https://doi.org/10.1021/bc900142y.

(10) Newkome, G. R.; Kotta, K. K.; Mishra, A.; Moorefield, C. N. Synthesis of Water-Soluble, Ester-Terminated Dendrons and Dendrimers Containing Internal PEG Linkages. *Macromolecules* **2004**, *37* (22), 8262–8268. https://doi.org/10.1021/ma049017i.

(11) Styslinger, T. J.; Zhang, N.; Bhatt, V. S.; Pettit, N.; Palmer, A. F.; Wang, P. G. Site-Selective Glycosylation of Hemoglobin with Variable Molecular Weight Oligosaccharides: A Potential Alternative to PEGylation. *J. Am. Chem. Soc.* **2012**, *134* (17), 7507–7515. https://doi.org/10.1021/ja300893t.
